# NLRP3 Inflammasome Priming and Activation Are Regulated by a Phosphatidylinositol-Dependent Mechanism

**DOI:** 10.4049/immunohorizons.2200058

**Published:** 2022-08-29

**Authors:** Claire Hamilton, Antoni Olona, Stuart Leishman, Kelly MacDonald-Ramsahai, Shamshad Cockcroft, Gerald Larrouy-Maumus, Paras K. Anand

**Affiliations:** * Department of Infectious Disease, Imperial College London, London, United Kingdom; † Department of Neuroscience, Physiology and Pharmacology, Division of Biosciences, University College London, W12 0NN London, United Kingdom; ‡ MRC Centre for Molecular Bacteriology and Infection, Department of Life Sciences, Imperial College London, London, United Kingdom

## Abstract

Imbalance in lipid homeostasis is associated with discrepancies in immune signaling and is tightly linked to metabolic disorders. The diverse ways in which lipids impact immune signaling, however, remain ambiguous. The phospholipid phosphatidylinositol (PI), which is implicated in numerous immune disorders, is chiefly defined by its phosphorylation status. By contrast, the significance of the two fatty acid chains attached to the PI remains unknown. In this study, by using a mass spectrometry–based assay, we demonstrate a role for PI acyl group chains in regulating both the priming and activation steps of the NOD-like receptor family pyrin domain-containing 3 (NLRP3) inflammasome in mouse macrophages. In response to NLRP3 stimuli, cells deficient in ABC transporter ATP Binding Cassette Subfamily B Member 1 (ABCB1), which effluxes lipid derivatives, revealed defective inflammasome activation. Mechanistically, Abcb1 deficiency shifted the total PI configuration exhibiting a reduced ratio of short-chain to long-chain PI acyl lipids. Consequently, Abcb1 deficiency initiated the rapid degradation of Toll/IL-1R domain–containing adaptor protein, the TLR adaptor protein that binds PI (4,5)-bisphosphate, resulting in defective TLR-dependent signaling, and thus NLRP3 expression. Moreover, this accompanied increased NLRP3 phosphorylation at the Ser291 position and contributed to blunted inflammasome activation. Exogenously supplementing wild-type cells with linoleic acid (LA), but not arachidonic acid, reconfigured PI acyl chains. Accordingly, LA supplementation increased Toll/IL-1R domain–containing adaptor protein degradation, elevated NLRP3 phosphorylation, and abrogated inflammasome activation. Furthermore, NLRP3 Ser291 phosphorylation was dependent on PGE2-induced protein kinase A signaling because pharmacological inhibition of this pathway in LA-enriched cells dephosphorylated NLRP3. Altogether, our study reveals, to our knowledge, a novel metabolic-inflammatory circuit that contributes to calibrating immune responses. *ImmunoHorizons*, 2022, 6: 642–659.

## Introduction

The NOD-like receptor family pyrin domain-containing 3 (NLRP3) inflammasome is a multiprotein complex activated in response to diverse pathogen-associated and “danger” signals; it plays important roles during infectious and inflammatory diseases (1–3). The activation of NLRP3 inflammasome involves two steps in which NLRP3 is first licensed downstream of TLR signaling. This step, also known as the priming step, has both NF-κB–dependent and -independent consequences during NLRP3 activation. Upon sensing an apt stimulus, NLRP3 complexes with pro-caspase-1 and the adaptor molecule apoptosis-associated speck-like protein containing a CARD (ASC). Consequently, caspase-1 is activated by autoproteolysis, which further results in the maturation and release of biologically active forms of cytokines IL-1β and IL-18. Additional regulation is mediated by the posttranslational modification of distinct NLRP3 domains, which may affect either the priming or activation of the NLRP3 inflammasome (4, 5). Inflammasome activation also results in the induction of an inflammatory form of cell death, pyroptosis, by a gasdermin-d (GSDMD)–dependent mechanism (6). Recent studies have revealed elaborate links between lipid metabolism and inflammasome activation (7). We, and others, previously demonstrated vital roles for cholesterol biosynthesis and transport in NLRP3 inflammasome activation (8, 9). Other recent studies have demonstrated that NLRP3 recruitment to the dispersed *trans*-Golgi network requires binding to phosphatidylinositol (PI) 4-phosphate prior to NLRP3 activation (10). The mechanisms of NLRP3 inflammasome activation and, remarkably, the roles lipids play in the process remain ambiguous.

Lipid homeostasis is critical to all physiological processes, including immune signaling (11). Lipids form the structural framework that imparts fluidity to membranes. Driven by their amphipathic nature, membrane lipids enable compartmentalization of cellular constituents both from the outside environment and into discrete organelles (12). The lipid composition of membranes, in addition, is pivotal in shaping the localization, conformation, and thus the activity of lipid–protein and protein-protein complexes (13). The latter is fundamental to cellular signaling emanating from the cholesterol-rich membrane-microdomains, which serve as signaling platforms and are the preferred sites for pathogen entry (14). In addition, phosphoinositides (PIPs), the phosphorylated derivatives of the parent PI, play important roles in immune signaling by ensuring precise recruitment of the adaptor protein Toll/IL-1R domain–containing adaptor protein (TIRAP), also known as Mal, to the activated TLRs at the plasma and endosomal membranes (15). Structurally, PI consists of an inositol head group and two fatty acyl chains linked by a glycerol backbone (16). The binding of PIPs to the PIP-binding domain of TIRAP results in the recruitment of MyD88 and the IL-1R–associated kinase (IRAK) family of kinases to activated TLRs, thereby promoting downstream NF-kB activation. Accordingly, the synthesis, turnover, and localization of PIPs may influence TLR-dependent signaling.

Homeostasis of the cellular lipid composition is largely maintained by the SREBP family of transcription factors that transcribe genes involved in lipid uptake, biosynthesis, and efflux (17). When in excess, lipids are either stored in the form of lipid droplets or are effluxed by the activity of the ABC family of transporters. The family members ABCA1 and ABCG1 facilitate cholesterol efflux to apolipoproteins and high-density lipoprotein, respectively (18). Moreover, deficiency in ABCA1 and ABCG1 is associated with enhanced secretion of inflammatory mediators, including IL-1β (19). Correspondingly, the efflux transporters play anti-inflammatory functions in diverse diseases (20, 21), suggesting a possible link between lipid metabolism and immune signaling (22). Cells deficient in *Abca1* and *Abcg1* cannot unload surplus lipids exhibiting elevated cholesterol accumulation, which independently improves cytokine secretion (18, 19). Overall, this argues that immune signaling is profoundly determined by lipid metabolism, but cholesterol accumulation remains a confounding factor, and detailed mechanisms remain poorly defined. In this study, we investigated the role of lipid metabolism in immune signaling by studying ATP Binding Cassette Subfamily B Member 1 (ABCB1). ABCB1 (or P-glycoprotein) is a well-characterized family member that imparts multidrug resistance to malignant cells but has no impact on cellular cholesterol levels (23, 24). The precise functions of ABCB1 in inflammasome activation remain undefined.

In this study, we demonstrate a role for ABCB1 and PI fatty acyl chains in regulating the NLRP3 inflammasome. Mechanistically, *Abcb1^–/–^* cells displayed a reduced ratio of short-chain to long-chain PI acyl chain lipids. This change in PI configuration was independent of the expression of enzymes that both synthesize PI and are involved in acyl chain remodeling. Remarkably, the shift in acyl chain composition regulated both NLRP3 priming and activation steps: it resulted in the depletion of TLR adaptor protein, TIRAP, and additionally elevated phosphorylation in the NAIP, CIITA, HET-E, and TP-1 (NACHT) domain of NLRP3. Intriguingly, exogenously supplementing wild-type (WT) cells with linoleic acid (LA) reconfigured PI acyl chains, which accordingly mimicked *Abcb1*-deficient cells in TIRAP depletion, NLRP3 phos-phorylation, and blunted inflammasome activity. Our study thus identifies an important role for PI lipid chain configuration in modulating inflammasome activity, which may have significant implications in metabolic diseases.

## Materials And Methods

### Ethics statement

Experiments involving animals were performed in accordance with the Animals (Scientific Procedures) Act 1986, in accordance with a current U.K. Home Office license, and with approval from the Imperial College Animal Welfare and Ethical Review Body.

### Bone marrow–derived macrophage isolation and cell culture

Bone marrow obtained from C57BL/6 mice (Charles River) was isolated from the femurs and tibias of 6- to 8-wk-old mice and cultured for macrophage differentiation as previously described (25, 26). WT immortalized bone marrow–derived macrophages (iBMDMs) were kindly provided by K. Fitzgerald and grown in DMEM containing 10% FBS, 1% penicillin/streptomycin, 1% HEPES, and 10% L929 conditioned medium. HEK293T cells were grown in DMEM containing 10% FBS and 1% HEPES at 37° C, 5% CO_2_. Cells were incubated and grown at 37° C, 5% CO_2_ and passaged every 2–3 d.

### Generation of CRISPR-Cas9 ABCB1b knockout cells

Guides targeting exons 10 and 11 of the Abcb1b gene were designed using the CHOPCHOP online software (http://chopchop.cbu.uib.no/) and the Zhang Lab CRISPR design software (http://crispr.mit.edu/). The guides contained a BsmBI overhang and were as follows: exon 10 guide RNA (gRNA), 5′-CACCGAAG CCTTTGCAAACGCACGA-3′, and its reverse complement, 5′-AA ACTCGTGCGTTTGCAAAGGCTTC-3′; and exon 11 gRNA, 5′-CA CCGCCCATCGAGAAGCGAAGTTC-3′, and its reverse complement, 5′-AAACGAACTTCGCTTCTCGATGGGC-3′. Guide RNAs were annealed and ligated into the LentiCRISPRv2 plasmid (52961; Addgene) at the *BsmBI* restriction site. The recombinant plasmids (LentiCRISPRv2.gRNA10, LentiCRISPRv2.gRNA11) were then transformed into competent *Escherichia coli* (NEB) according to the manufacturer’s instructions. A total of 1.85 μg LentiCRISPRv2gRNA10 plasmid or LentiCRISPRv2gRNA10 plasmid, 0.42 μg pVSVg plasmid, and 1.3 μg psPAX2 plasmid were together transfected into the HEK923T cells using polyethyleneimine (Sigma), at a ratio of 1 μg DNA:3 μg polyethyleneimine. Supernatants containing lentiviral particles from transfected HEK293T cells were harvested at 48 h posttransfection. A total of 300 μl of lentiviral particle-containing supernatant was then added to the iBMDMs, and cells were incubated at 37° C for ~16 h after which fresh media were added to cells. Forty-eight hours after transduction, puromycin (6 μg/ml; Sigma) was added for 7–10 d to select cells that had been successfully transduced. Puromycin-resistant cells were then trypsinized and seeded into a 96-well plate at an approximate concentration of one cell per well to obtain a clonal cell population. Single-cell colonies were identified and expanded followed by Sanger sequencing to identify mutations in exons 10 or 11 of the *Abcb1b* gene. Two clones, *Abcb1b^–/–^* #1 and #2, were identified with deletions of 32 and 9 bp in exon 11, respectively (Supplemental Fig. 2).

### Cell stimulations

Primary and immortalized macrophages were seeded into either 6-well plates at a concentration of 2.5 × 10^6^, 12-well plates at a concentration of 1 × 10^6^, or 24-well plates at a concentration of 0.5 × 10^6^ cells/well. Where indicated, macrophages were treated with elacridar (1–10 μM, SML0486; Sigma) for 16 h. In experiments with other inhibitors, methyl-β-cyclodextrin (5–10 μM, C4555; Sigma) or MG132 (10 μM) was added 30 min before the addition of LPS. In other experiments, cells were exposed to IL-1 (2 ng/ml, 211-11B; PeproTech), cyclooxygenase 2 (COX2) inhibitor NS-398 (10 μM, 70590; Cayman), or protein kinase A (PKA) inhibitor H-89 (10 μM, 10010556; Cayman) overnight. For fatty acid supplementation, distinct fatty acids were conjugated to albumin as previously described (27). Cells were continuously cultured with albumin-bound arachidonic acid (AA; 5 μM), LA (20 μM), or a combination of AA (5 μM) and stearic acid (SA; 20 μM) for at least 2 wk before mass spectrometry (MS) analysis or any further experiments.

### Inflammasome activation

To activate the NLRP3 inflammasome, we incubated cells with LPS (500 ng/ml; Invivogen) for 4–6 h to prime the macrophages, followed by ATP (0.5 μM; Sigma) or nigericin (20 μM; Tocris) for ~45 min. For NLRC4 inflammasome activation, the *Salmonella Typhimurium* strain SL1344 was cultured overnight in 5 ml Luria-Bertani broth at 37° C and on a shaker at 220 rpm. The bacteria were then added to the indicated cells, treated with or without elacridar (1–5 μM) or *Abcb1b^–/–^* cells, at a multiplicity of infection (MOI) of 2, and incubated for ~4 h. For AIM2 inflammasome activation, macrophages were transfected with 1 μg of poly(deoxyadenylic-deoxythymidylic) acid sodium salt [poly(dA:dT)] (Invivogen) complexed with Lipofectamine 2000 at a 1:3 ratio according to the manufacturer’s instructions for ~4–5 h.

### Cell signaling experiments

The indicated cells were seeded into six-well plates at a concentration of 2 × 10^6^ cells/well and incubated with elacridar (5 μM) overnight. The following morning, media were replaced, and cells were stimulated with LPS (500 ng/ml) for the following time points: 0 (no LPS control), 0.5, 1, 2 and 4 h. Supernatants were discarded from each well, the cells were subsequently washed with PBS, and cells were collected in ra-dioimmunoprecipitation buffer. Samples were incubated on ice for ~30 min before being centrifuged at 15,000 × *g* for 15 min at 4°C to remove nuclei. The supernatant was collected, and the protein concentration of each sample was measured using the BCA Protein Assay kit (23227; Thermo Scientific) according to the manufacturer’s instructions. Samples were all standardized to 1 μg/μl before immunoblot analysis.

### Immunoblot analysis

For immunoblot of phospho-specific Abs, cells were collected in radioimmunoprecipitation lysis buffer containing both protease and phosphatase inhibitors (Roche) and standardized to 1 μg/μl as mentioned earlier. Samples were boiled at 95° C for 5 min before being resolved on 12% SDS-PAGE gels. For immunoblotting of caspase-1, NLRP3, IL-1β, ASC, GSDMD, and GAPDH, lysates were collected in cell lysis buffer containing Nonidet P-40, DTT, and protease inhibitors. Samples were boiled at 95°C for ~20–30 min before being resolved on 12% SDS-PAGE gels. SDS-PAGE gels were transferred to nitrocellulose membranes (GE Life Sciences) and subsequently blocked in a 5% milk solution in TBS-Tween (0.05%). Membranes were then incubated with the primary Ab overnight at 4° C followed by incubation with the HRP-conjugated secondary Ab at room temperature for 1 h. The primary Abs used were as follows: P-glycoprotein (1:1000, MA1-2652; Invitrogen), caspase-1 (1:2000, AG-20B-0042-C100; AdipoGen), NLRP3 (1:2000, AG-20B-0014-C100; AdipoGen), ASC (1:2000, AG-25B-0006, AL177; AdipoGen), GSDMD (1:1000, ab209845; Abcam), IL-1β (1:500, 12426; Cell Signaling Technology), GAPDH (1:2500, MA5-15738; Thermo Fisher), Ikba (1:1000, 9242; Cell Signaling Technology), p-IKba (1:1000, 2859S; Cell Signaling Technology), p38 MAPK (1:1000, 9212; Cell Signaling Technology), p-p38 MAPK (1:1000, 4511; Cell Signaling Technology), AKT (1:1,000; 4691; Cell Signaling Technology), p-AKT (1:1,000; 4060; Cell Signaling Technology), p70-S6K1 (1:1,000; 2708; Cell Signaling Technology), p-p70-S6K1 (1:1,000; 9234; Cell Signaling Technology), TIRAP (1:1000; #PA5-88657; Thermo Fisher), and p-NLRP3 (1:1000, PA5-105071; Thermo Fisher). The HRP-conjugated secondary Abs (Thermo Fisher) were used at 1:5000. After secondary Ab incubation, proteins were visualized using either Bio-Rad Clarity ECL substrate (1705060; Bio-Rad) or the Pierce ECL Western blotting Substrate (32209; Thermo Scientific) and processed on a Bio-Rad imager. Images were obtained using the BioRad software, ImageJ.

### ELISA

Cell culture supernatants were measured for IL-1β (88-7013-88; eBioscience), TNF-α (88-7324-88; eBioscience), and IL-18 (7625; MBL), using ELISA kits according to the manufacturer’s instructions.

### Real-time PCR

RNA was isolated using TRIzol (T9424; Sigma) according to the manufacturer’s instructions. A total of 250 μg of RNA from each sample was then reverse transcribed into cDNA using the High-capacity cDNA Reverse Transcription kit (4368814; Applied Biosystems) according to the manufacturer’s instructions. Real-time PCR was then performed using the specific primers detailed in Supplemental Fig. 1A. Real-time PCR was performed on an ABI7500 or ABI7900HT (Applied Biosystems) fast real-time PCR instrument.

### Rhodamine-123 accumulation assay

BMDMs or *Abcb1b^–/–^* clones were seeded into 96-well plates at a concentration of 1 × 10^5^ cells per well. Where indicated, BMDMs were treated with elacridar overnight (2–10 μM). Rhodamine-123 (Rho123; 2 μM, Sigma) was added, and cells were incubated at 37° C for 30 min. Cells were then washed with PBS three times to remove any extracellular Rho123, and DMEM were replaced, after which cells were incubated for a further 30 min at 37° C. Cells were then washed again with PBS and lysed in cell lysis buffer before being read at excitation and emission wavelengths of 485 and 535 nm, respectively, in a fluorescent plate reader.

### Total cholesterol measurement

Total cholesterol was measured using the Cholesterol Amplex Assay kit according to the manufacturer’s recommendations.

### Filipin staining

WT and *Abcb1b^–/–^* cells were seeded onto coverslips at a concentration of 4 × 10^5^ cells/well. After they had adhered, cells were fixed in 4% paraformaldehyde for 1 h at room temperature. Cells were then stained with 25 μg/ml filipin (F9765; Sigma) overnight and washed three times with PBS before mounting on glass slides. Images were visualized on a Leica SP5 confocal microscope using 405 nm excitation and processed using the ImageJ program.

### GM1 staining

Lipid rafts were assessed by specific labeling of endogenous GM1 ganglioside (a lipid raft marker) with the Vybrant Alexa Fluor 488 Lipid Raft labeling kit, which makes use of fluorescently conjugated cholera toxin subunit B (CTB). The procedure was carried out according to the manufacturer’s instructions (Thermo Fisher). Samples were imaged using a Leica SP5 confocal microscope. Flow cytometry was performed using the Attune NxT Flow Cytometer (Thermo Fisher), and mean fluorescence intensity was calculated using FlowJo v10.

### Caspase-1 inflammasome assay

In some experiments, caspase-1 activity in the cell culture supernatants was measured using Caspase-Glo 1 inflammasome assay according to the manufacturer’s instructions (Promega). Luminescence was measured using an Omega plate reader.

### Immunofluorescence

WT, *Abcb1b^–/–^*, and LA-supplemented cells were seeded onto coverslips at a concentration of 4 × 10^5^ cells/well. After the ex-perimental conditions, cells were fixed in 4% paraformaldehyde for 1 h at room temperature and washed twice with PBS-glycine (50 mM). The coverslips were blocked by incubating them for 20 min with PBS containing 1% BSA. Cells were then labeled with either anti-ASC Ab (1:100; AG-25B-0006, AL177; AdipoGen) or anti-TIRAP Ab (1:100; #PA5-88657; Thermo Fisher) for 1 h at room temperature. Coverslips were then washed with PBS twice before incubating them with secondary Abs (Thermo Fisher). Actin staining was carried out by labeling samples with Alexa Fluor 647–conjugated phalloidin (A22287; Thermo Fisher) for 30 min. Coverslips were washed three times and mounted on slides using Fluoromount-G mounting medium with DAPI (00-4959-52; Thermo Fisher). Images were acquired on an SP5 confocal microscope and analyzed using ImageJ software.

### MALDI-MS analysis

WT and *Abcb1b^–/–^* macrophages were cultured in DMEM before being trypsinized and analyzed via MS. Before analysis, the super-2,5-dihydroxybenzoic acid (catalog no. 50862; Sigma-Aldrich) matrix was added at a concentration of 10 mg/ml in a chloroform/methanol mixture at a 90:10 (v/v) ratio; 0.4 mlofa cell solution at a concentration of 2 × 10^5^/ml to 2 × 10^6^/ml was preliminary washed three times with double-distilled water, corresponding to ~100–1000 cells/well of the MALDI target plate (384 Opti-TOF 123 mm × 84 mm AB Sciex NC0318050, 1016629); and 0.8 μl of the matrix solution was deposited on the MALDI target plate. These were then gently mixed with a micropipette and left to dry. MALDI-TOF MS analyses were performed on a 4800 Proteomics Analyzer (with TOF-TOF Optics; Applied Biosystems) using the reflectron mode. Samples were analyzed in the negative ion mode operating at 20 kV. A total of three independent experiments were performed. Data obtained from MS were analyzed using Data Explorer version 4.9 (Applied Biosystems), and assignments were based on the MS/MS fragmentation profile.

### Statistical analysis

GraphPad Prism 9.0 software was used for data analysis. Data are represented as mean ± SD and are representative of experiments done at least three times. Statistical significance was determined by unpaired Student *t* test; *p* < 0.05 was considered statistically significant.

## Results

### ABC transporter B family member 1 is required for caspase-1 activation and IL-1β secretion

Lipids are involved in diverse functions, including maintenance of membrane structure, cellular signaling, and immunity (11, 12). Consequently, altered lipid levels can lead to metabolic and inflammatory disorders or may result in lipotoxicity (20). Therefore, lipid homeostasis, and in particular, lipid efflux, is critical to normal cell function. Members of the ABC family function to export substrates, mainly lipids and related molecules, out of the cytosol (18). For example, ABCA1 is primarily associated with phospholipid and cholesterol transport to lipid-poor apolipoprotein A-I, while ABCG1 exports cholesterol to more mature high-density lipoprotein particles. Remarkably, deficiency in *Abca1* and *Abcg1* results in elevated secretion of inflammatory mediators in response to pathogenic stimuli (19). To address the roles of lipid metabolism in immune signaling, we investigated the role of ABCB1 in inflammasome activation. ABCB1 alters lipid metabolism and has a broad substrate specificity in transporting a range of drugs (28). However, the transporter does not affect overall cellular lipid or cholesterol levels.

To test the role ABCB1 plays in inflammasome activation, we generated genetic knockout cell lines using the CRISPR-Cas9 approach in iBMDMs. In contrast with humans where a single isoform of ABCB1 is expressed, the mouse genome To test the activity of ABCB1 in edited cells expresses two isoforms, *Abcb1a* and *Abcb1b* (29). We first examined the expression of the two isoforms in bone marrow-derived mouse macrophages. PCR amplification followed by gel electrophoresis revealed that both *Abcb1a* and *Abcb1b* are expressed in unstimulated and LPS-stimulated iBMDMs (Supplemental Fig. 1B, 1C). However, quantitative PCR revealed that *Abcb1b* is expressed at ~600-fold higher levels than that of *Abcb1a*, suggesting that this isoform predominates in mouse macrophages (Supplemental Fig. 1D). Similar results were obtained with primary mouse macrophages (Supplemental Fig. 1E; data not shown). In addition, the expression of *Abcb1b* was only marginally higher in LPS-stimulated macrophages compared with control cells at all time points tested, although there was a slight initial decrease in expression on LPS stimulation (Supplemental Fig. 1F). These studies therefore suggest a potential role for ABCB1 in immune cells.

**Figure 1:**
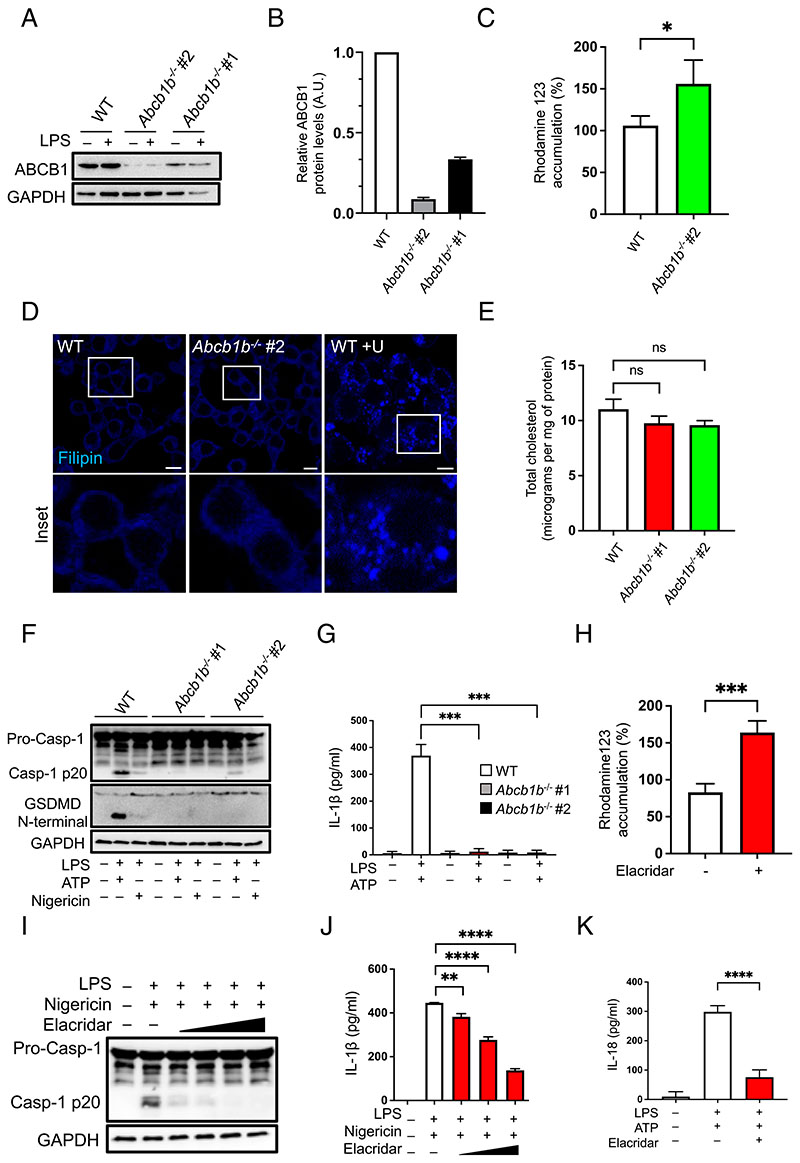
ABC transporter B family member 1 is required for caspase-1 activation and IL-1β secretion. (**A**) Cell lysates from WT and *Abcb1b^–/–^* #1 and #2 cells were treated with or without LPS (500 ng/ml) for 4 h and immunoblotted for ABCB1 and GAPDH. (**B**) Relative ABCB1 protein levels in WT and *Abcb1b^–/–^* #1 and #2 cells measured with ImageJ on blots shown in (A). (**C**) WT and *Abcb1b^–/–^* #1 and #2 cells were incubated with Rho123 (1 μM) for 30 min before measuring Rho123 accumulation on a fluorescent plate reader. (**D**) WT cells without and with overnight exposure to U18666a (5 μg/ml) and control *Abcb1b^–/–^* cells grown on coverslips were fixed in paraformaldehyde for 1 h followed by overnight staining with filipin (25 μg/ml) at 4°C. Images were taken by confocal microscopy. (**E**) Total cholesterol content in WT and *Abcb1b^–/–^* #1 and #2 cells measured by Amplex red assay. (**F**) WT, *Abcb1b^–/–^* #1, and *Abcb1b^–/–^* #2 cells were primed with LPS (500 ng/ml) for 4 h followed by treatment with either ATP (5 mM) or nigericin (20 μM) for ~45 min. Cell lysates were collected and immunoblotted for caspase-1, GSDMD, and GAPDH. (**G**) Supernatants from macrophages treated as in (F) were analyzed for IL-1β secretion by ELISA. (**H**) Primary mouse BMDMs were exposed to elacridar overnight (10 μM) and incubated with Rho123 (1 μM) for 30 min to evaluate Rh123 accumulation as in (C). (**I**) BMDMs were treated with increasing amounts of elacridar overnight (1, 2, 5, and 10 μM), followed by LPS priming (500 ng/ml) for 4 h and nigericin (20 μM) for ~45 min. Cell lysates were immunoblotted for caspase-1 and GAPDH. (**J**) Supernatants from cells treated as in (I) were analyzed for IL-1β secretion by ELISA. (**K**) iBMDMs were treated with elacridar overnight (10 μM) followed by LPS priming (500 ng/ml) for 4 h and ATP (5 mM) for ~45 min. Cell supernatants were analyzed for IL-18 secretion by ELISA. Data shown are mean ± SD, and the experiments shown are representative of at least three independent experiments with three to five replicates each. *p < 0.05, **p < 0.01, ***p < 0.001, ****p < 0.0001, by Student *t* test.

Two cell lines, *Abcb1b^–/–^* #1 and #2, were established with deletions in exon 10 of the protein, as verified by Sanger sequencing (Supplemental Fig. 1G). The mRNA expression of *Abcb1b* in the two CRISPR cell lines was reduced significantly, although the PCR amplicon was still synthesized to some extent (Supplemental Fig. 1H). However, compared with WT cells, the protein expression of ABCB1 was diminished by >70% and >90% in the two cell lines, potentially suggesting the presence of unstable mRNA in edited cell lines (Fig. 1A, 1B).

To test the activity of ABCB1 in edited cells, we made use of Rho123 dye. Rho123 passively diffuses across biological membranes but is subsequently metabolized by intracellular esterases to yield a fluorescent compound that has reduced permeability (30). The efflux of the dye out of cells requires active transport by ABCB1 and certain other ABC transporters (30). *Abcb1^–/–^* cells exposed for 30 min to Rho123 showed increased sequestration of the dye compared with control cells, thereby confirming inhibition of ABCB1 transporter activity (Fig. 1C).

Unlike other ABC transporters that are involved in lipid efflux, ABCB1 is not known to alter cellular cholesterol levels (31). In agreement, WT and *Abcb1^–/–^* macrophages exhibited no qualitative differences in cholesterol distribution on staining with filipin, a compound that binds to free unesterified cholesterol (8). Excitation of filipin by UV fluorescence showed no obvious differences in either the levels or distribution of cholesterol between WT and *Abcb1^–/–^* cells (Fig. 1D). As a control, we exposed WT cells to U18666a, which specifically blocks the lysosomal cholesterol transporter, NPC1 (8), and this resulted in expected punctate filipin staining suggestive of lysosomal cholesterol accumulation (Fig. 1D). In addition, quantitative analysis of total cellular cholesterol revealed similar levels in both WT and *Abcb1^–/–^* cells (Fig. 1E). These data agree with previous studies and indicate no major effect of ABCB1 deficiency on cholesterol esterification, distribution, and efflux.

As summarized earlier, the roles of ABCB1 in inflammasome activation and IL-1β secretion remain unknown. To address this, we next tested the activation of NLRP3 inflammasome in macrophages with genetic deletion of *Abcb1b*. WT and *Abcb1^–/–^* cells were exposed to LPS for 4 h followed by either ATP or nigericin. NLRP3 inflammasome activation results in the assembly of the inflammasome complex containing NLRP3, the adaptor ASC, and pro-caspase-1. Pro-caspase-1 is then cleaved into its active p20 form, which subsequently results in the maturation and secretion of proinflammatory cyto-kines, IL-1β and IL-18. Compared with WT cells, *Abcb1^–/–^* cells exhibited diminished caspase-1 cleavage and IL-1β secretion on exposure to both nigericin and ATP (Fig. 1F, 1G). Activation of the NLRP3 inflammasome also leads to an inflammatory form of cell death, termed pyroptosis, which is mediated by GSDMD. Cleavage of GSDMD by caspase-1 results in an N-terminal fragment, which subsequently polymerizes and forms pores in the cell membrane, ultimately leading to cell rupture. GSDMD cleavage in *Abcb1b^–/–^* macrophages was diminished in comparison with WT cells after NLRP3 inflammasome activation, further corroborating reduced caspase-1 in deficient cells (Fig. 1F).

We next used elacridar, a third-generation inhibitor that has been shown to inhibit ABCB1 activity and overcome drug resistance in cancer models (32). Primary BMDMs treated overnight with increasing concentrations of elacridar and exposed for 30 min to Rho123 exhibited increased sequestration of the dye compared with control cells (Fig. 1H). In agreement with deficient cells, treatment of WT macrophages with increasing concentrations of elacridar followed by NLRP3 activation resulted in a dose-dependent decrease in the generation of cleaved caspase-1 p20 form (Fig. 1I) and reduction in IL-1β and IL-18 secretion (Fig. 1J, 1K). Similar results were observed with the P2X_7_ receptor agonist and NLRP3 activator, ATP (Supplemental Fig. 2A, 2B, Fig. 1K). Together, these data demonstrate the functional requirement of ABCB1 for caspase-1 activation and IL-1β release.

### ABCB1 regulates NF-κB-dependent signaling and priming of the NLRP3 inflammasome

**Figure 2:**
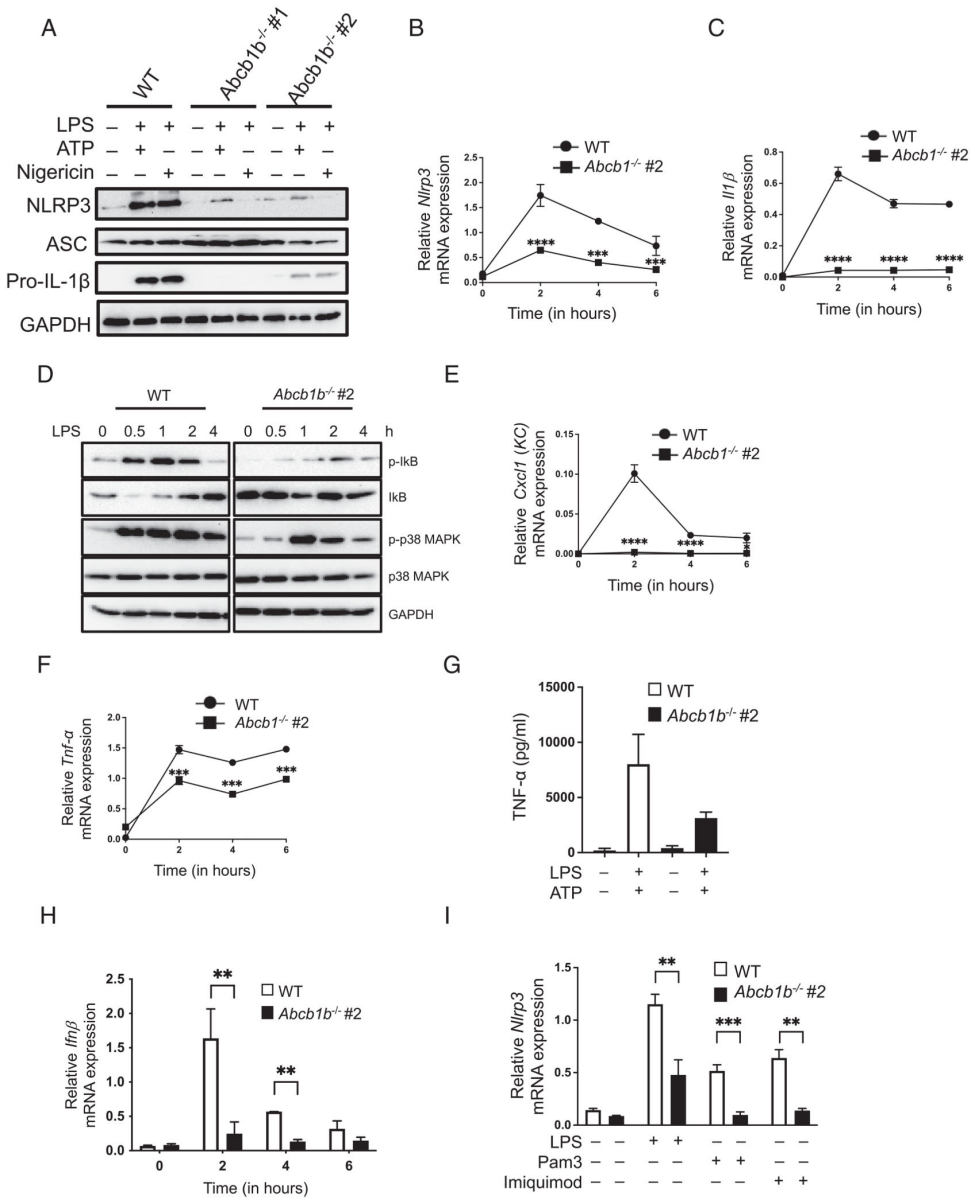
ABCB1 regulates NF-κB–dependent signaling and priming of the NLRP3 inflammasome. (**A**) WT and *Abcb1b^–/–^* #1 and #2 cells primed with LPS (500 ng/ml) for 4 h followed by treatment with either ATP (5 mM) or nigericin (20 μM) for ~45 min. Cell lysates were collected and immunoblotted for NLRP3, ASC, pro-IL-1β, and GAPDH. (**B**) WT and *Abcb1b^–/–^* #2 cells were stimulated with LPS (500 ng/ml) for the indicated time points. RNA was extracted and converted into cDNA. Gene expression of *Nlrp3* and (**C**) *Il1b* was determined by real-time PCR. (**D**) WT and *Abcb1b^–/–^* #2 cells were stimulated with LPS (500 ng/ml) for the indicated time points. Protein samples were collected and immunoblotted for p-IκB, total IκB, p-p38 MAPK, total p38 MAPK, and GAPDH. (**E**) Cells treated as in (B) were examined for *Cxcll* (*KC*) and (**F**) *Tnfα* gene expression by real-time PCR. (**G**) Supernatants from WT and *Abcb1b^–/–^* #2 macrophages treated as in (B) were analyzed for TNF-*α* cytokine secretion by ELISA. (**H**) WT and *Abcb1b^–/–^* #2 cells were stimulated with LPS (500 ng/ml) for 4 h and analyzed for mRNA expression of *Ifnβ*. mRNA expression is shown relative to *Gapdh*. (**I**) WT and *Abcb1b^–/–^* #2 cells were stimulated with either LPS (500 ng/ml), Pam3 (1 μg/ml), or Imiquimod (1 μg/ml) for 4 h and analyzed for *Nlrp3* mRNA expression. Gene expression is shown relative to *Gapdh*. Data shown are mean ± SD, and the experiments shown are representative of three independent experiments with three replicates each. **p < 0.01, ***p < 0.001, ****p < 0.0001, by Student *t* test.

NLRP3 inflammasome activation requires two distinct steps. The priming step results in the induction of TLR-dependent NF-κB activation, which in turn leads to the transcriptional and translational upregulation of NLRP3 and pro–IL-1β. The second signal activates the NLRP3 inflammasome complex, leading to caspase-1 activation (33, 34). We next studied the precise step at which ABCB1 is required for activation of the NLRP3 inflammasome by examining the expression of inflammasome components, including NLRP3 and pro-IL-1β. Stimulation with LPS and ATP upregulated the NLRP3 and pro-IL-1β protein in WT cells, which was found to be blunted in cells lacking *Abcb1* (Fig. 2A). These results coincided with reduced upregulation of *Nlrp3* and *Illb* at the mRNA level (Fig. 2B, 2C). By contrast, pro-caspase-1 and ASC are constitutively expressed, and their expression remained unaltered in *Abcb1^–/–^* macrophages (Figs. 1F, 2A, Supplemental Fig. 2C, 2D).

NF-κB is inactive within the cell before stimulation through binding to the inhibitory protein IκB. On TLR stimulation, signal transduction leads to the activation of the IκB kinase complex, which subsequently phosphorylates IκB, targeting it for degradation. This allows NF-κB to translocate to the nucleus and initiate gene transcription. In addition to NF-κB activation, TLR stimulation also leads to the activation of MAPKs, notably p38 (35). In agreement with the data shown in Fig. 2A-C, the phosphorylation of IkB and p38 MAPK was reduced in LPS-stimulated *Abcb1^–/–^* cells compared with WT cells (Fig. 2D). We also examined the expression of other NF-κB-dependent cytokines and chemokines in response to TLR4 activation, and their expressions were similarly decreased in cells lacking ABCB1 (Fig. 2E-G). Moreover, the mRNA expression of IFN-β, which is dependent on adaptor TRIF on LPS stimulation, was also reduced in *Abcb1^–/–^* cells (Fig. 2H). Furthermore, this response was not specific to TLR4 ligation, because activation of TLR2 or TLR7 by Pam3CSK4 and imiquimod, respectively, also resulted in diminished upregulation of *Nlrp3*, *Illb*, *Tnfα*, and *Cxcll* (KC) mRNA expression (Fig. 2I, Supplemental Fig. 2E-G). These data suggested blunted TLR-dependent NF-kB signaling in the absence of ABCB1.

### ABCB1 does not affect the activation of NLRC4 and AIM2 inflammasomes

**Figure 3:**
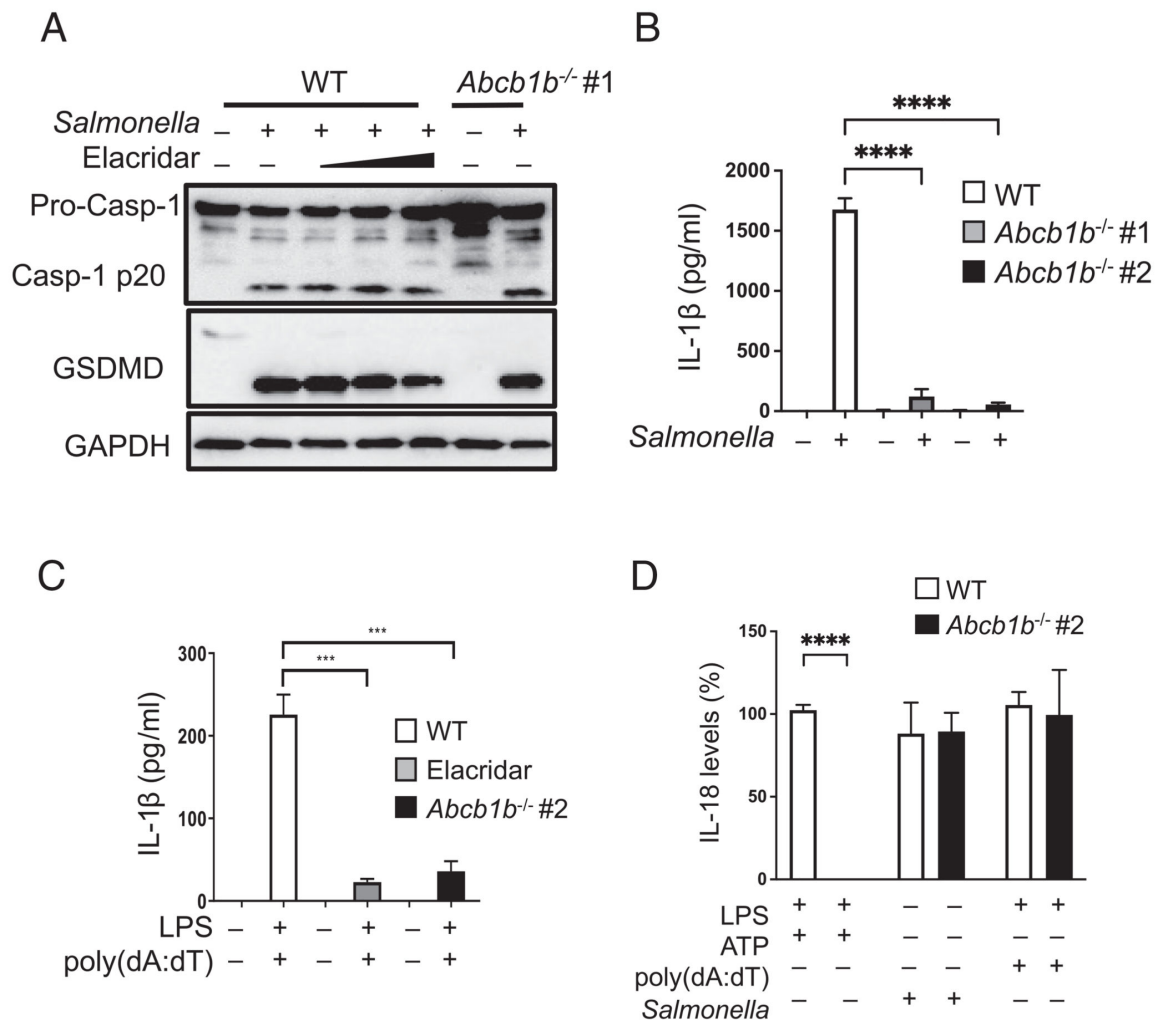
ABCB1 does not affect the activation of NLRC4 and AIM2 inflammasomes. (**A**) WT cells with or without exposure to elacridar (2, 5, 10 μM; 16 h) and *Abcb1b^–/–^* #1 cells were infected with *Salmonella typhimurium* at an MOI of 2 for ~4–5 h. Cell lysates were collected and immunoblotted for caspase-1, GSDMD, and GAPDH. (**B**) WT, *Abcb1b^–/–^* #1, and *Abcb1b^–/–^* #2 macrophages were infected with *Salmonella typhimurium* at an MOI of 2 for ~4-5 h, and supernatants were analyzed for IL-1β by ELISA. (**C**) Cells were treated with LPS (500 ng/ml) for 4 h followed by transfection with 1 μg of DNA complexed to Lipofectamine 2000 (ratio DNA:Li-pofectamine 2000, 1:3) for ~4 h to activate the AIM2 inflammasome. Supernatants were analyzed for IL-1β by ELISA. (**D**) Supernatants from cells treated as in (B) and (C) or treated with LPS (500 ng/ml, 4 h) and ATP (5 mM, 45 min) were analyzed for IL-18 production by ELISA. Data shown are mean ± SD and are representative of at least three independent experiments with three replicates each. ***p < 0.001, ****p < 0.0001, by Student *t* test.

We next tested the requirement of ABCB1 during NLRC4 and AIM2 inflammasome activation. Unlike NLRP3, the expressions of NLRC4 and AIM2 are constitutively expressed at significantly higher levels in mouse macrophages. However, the expression and therefore the secretion of downstream effector cytokine IL-1β still require upregulation by TLR signaling. Activation of NLRC4 inflammasome by *Salmonella* infection and AIM2 inflammasome by exposure to poly(dA:dT) resulted in comparable caspase-1 cleavage between WT and *Abcb1b^–/–^* cells (Fig. 3A; data not shown). However, the levels of IL-1β secretion, as expected, were found diminished in both *Abcb1^–/–^* cells and WT cells, where ABCB1 was pharmacologically blocked during NLRC4 and AIM2 inflammasome activation (Fig. 3B, 3C). In contrast with pro-IL-1β, pro-IL-18 is constitutively expressed and does not require TLR signaling for upregulation. In agreement, no difference in IL-18 production was observed after NLRC4 or AIM2 inflammasome activation in cells lacking ABCB1 (Fig. 3D). Secretion of IL-18, however, after NLRP3 activation showed complete abolishment in *Abcb1b^–/–^* macrophages (Fig. 3D). These data suggest that the function of ABCB1 is specific to the NLRP3 inflammasome, and that the diminished IL-1β production is due to dampened NF-kB signaling in *Abcb1b^–/–^* cells (Fig. 2C, 2D).

### ABCB1 deficiency is associated with a shift in PI lipid chains

A notable feature of the plasma membrane is the distinct lipid composition in the two leaflets of the bilayer (36). Sphingolipids are mostly present in the outer leaflet, while glycerophospholipids, such as phosphatidylethanolamine, phosphatidylserine, and PI, are mostly restricted to the inner leaflet, thereby imparting distinct functions to the membranes (36).

**Figure 4:**
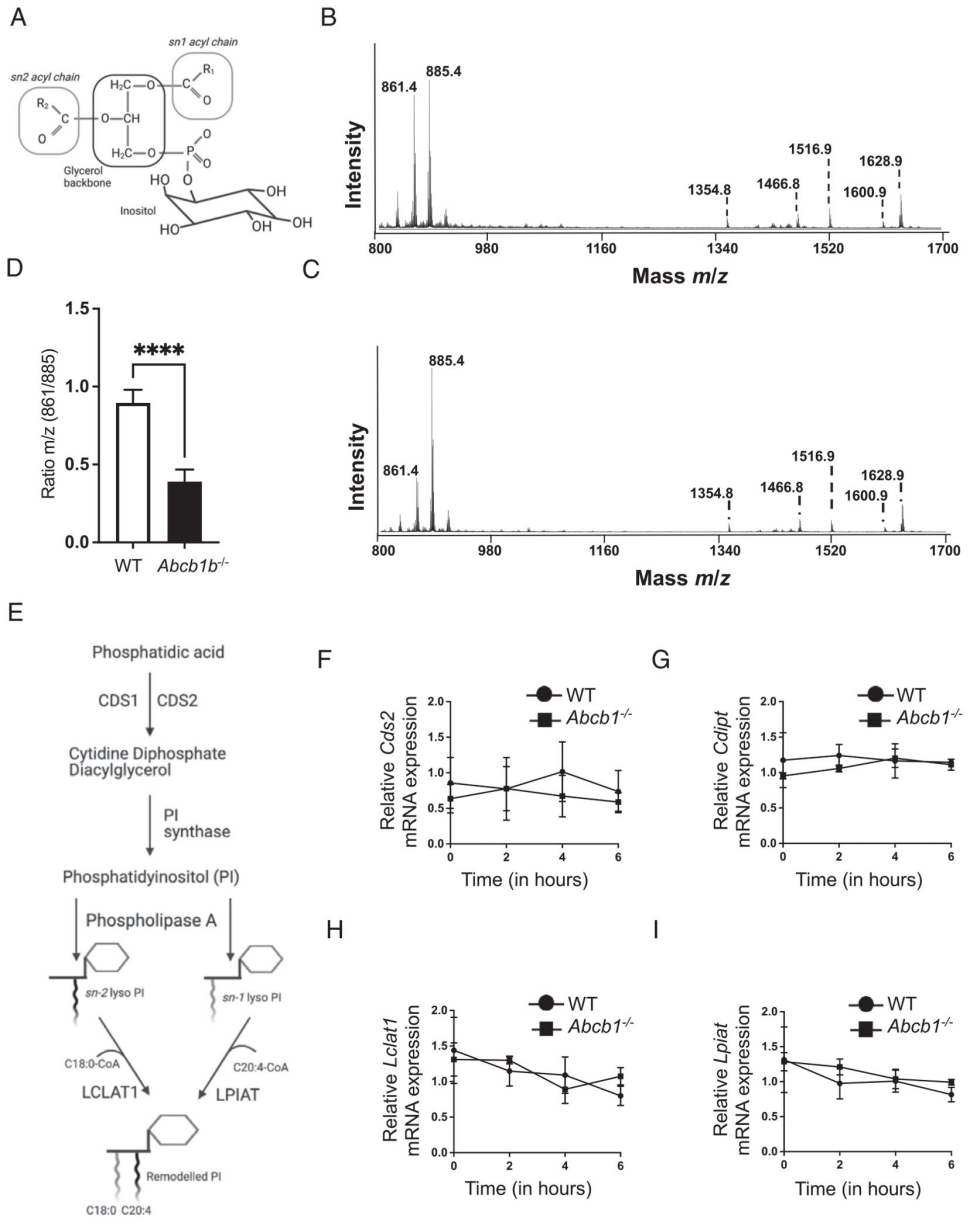
ABCB1 deficiency is associated with a shift in PI lipid chains. (**A**) PI structure consists of an inositol headgroup, a glycerol backbone, and two acyl chains R_1_ and R_2_ at the *sn-1* and *sn-2* positions. Representative spectra of (**B**) WT and (**C**) *Abcb1b^–/–^* cells. Peaks of interest are indicated. The peaks at *m/z* 835.4, 861.4, 885.4, and 911.5 are assigned to PI 34:1, 36:2, 38:4, and 40:4, respectively. In this range are also found peaks at *m/z* 1,354.7, *m/z* 1,438.8, and *m/z* 1,466.8, which are assigned to GM-2 d18:1/16:0, GM-2 d18:1/22:0, and GM-2 d18:1/C24:0, respectively. In the range *m/z* 1,500-1,650 are found GM-1 at *m/z* 1,516.8, *m/z* 1,544.8, *m/z* 1,572.8, *m/z* 1,600.9, *m/z* 1,626.9, and *m/z* 1,628.9 assigned to GM-1 18:1/16:0, 18:1/18:0, 18:1/20:0, 18:1/22:0, 18:1/24:1, and 18:1/24:0, respectively. (**D**) Ratio *m/z* of peaks 861/885 in WT and *Abcb1b^–/–^* macrophages. (**E**) Model showing PI synthesis and subsequent remodeling to acquire typical fatty acid configuration. WT and *Abcb1b^–/–^* #2 cells were treated with LPS (500 ng/ml) for the indicated time points, and the mRNA expressions of (**F**) *Cds2*, (**G**) *Cdipt*, (**H**) *Lclat1*, and (I) *Il1b* were analyzed. Gene expression is shown relative to *Gapdh*. Data shown are mean ± SD and are representative of at least three independent experiments with three replicates each. ****p < 0.0001, by Student *t* test.

To further investigate the mechanism by which *Abcb1* deficiency dampens NLRP3 inflammasome, we performed wholecell lipidomics by MALTI-TOF MS. Our experiments revealed ions in the 800–1700 mass to charge ratio (*m/z*) range. The most striking change was observed in the 800–920 *m/z* range, which represents the total PI complement present in cells with distinct signals corresponding to anticipated PI masses. In mammalian cells, a large fraction of the PIP molecules have saturated SA (18:0) at the *sn*-1 position and unsaturated AA (20:4) at the *sn*-2 position (Fig. 4A). Consequently, both the WT and *Abcb1^–/–^* cells exhibited a peak at *m/z* 885.4, described together as PI (38:4) (Fig. 4B, 4C). In addition, another PI peak at *m/z* 861.4, corresponding to PI (36:2), was observed in WT and *Abcb1^–/–^* cells (Fig. 4B, 4C). MS/MS fragmentation of the peaks at *m/z* 861 and *m/z* 885 revealed the presence of SA, AA, and oleic acid (Supplemental Fig. 3A, 3B). Besides the presence of PIs with distinct acyl chain lengths, other closely related PI siblings were also represented, although at much lower levels (Fig. 4B, 4C, Supplemental Fig. 3C). Notably, the WT cells exhibited almost equal levels of the two PI-lipid masses at 861 and 885. By contrast, *Abcb1*-deficient cells favored higher levels of PI (38:4) compared with PI (36:2), which was expressed at significantly lower levels. As a result, the ratio of the two PI-lipid masses consistently demonstrated a significant difference between WT and *Abcb1^–/–^* cells (Fig. 4D). These studies therefore suggest that the deficiency in *Abcb1* modifies the PI fatty acyl configuration, resulting in a reduced ratio of short-chain to long-chain fatty acids (Fig. 4D).

Further analysis of the MS data also unveiled several ganglioside peaks that were distinct between WT and *Abcb1b^–/–^* macrophages. In particular, the percentage of GM1 ganglioside was significantly reduced in *Abcb1b^-^/^-^* cells (Supplemental Fig. 4A), which was further confirmed qualitatively and quantitatively by labeling WT and *Abcb1^–/–^* cells with CTB or by flow cytometry (Supplemental Fig. 4B-F).

The de novo synthesis of PI occurs at the endoplasmic reticulum through the conversion of phosphatidic acid (PA) via two enzymatic reactions (Fig. 4E). PA is first converted into an intermediate, cytidine diphosphate-diacylglycerol (CDP-DAG), by CDP-DAG synthase. CDP-DAG is then converted to PI by PI-synthase (37, 38). Cleavage of the fatty acid by phospholipases A, followed by acylation by acyl-CoA-specific lysophospholipid acyltransferase enzymes, is needed to incorporate a new fatty acid molecule (39). Lysophosphatidylinositol acyltransferase has been shown to reacylate PI with polyunsaturated substrates such as AA, while lysocardiolipin acyltransferase 1 has been demonstrated to reacylate with SA (39) (Fig. 4E). Because *Abcb1b*-deficient macrophages displayed altered PI lipid chains, we investigated whether this was due to a defect in PI synthesis and/or acyl chain remodeling. However, the mRNA expression of *Cds2* (*Cds1* was expressed with cycle threshold value > 30) and *Cdipt* revealed no significant difference between WT and *Abcb1b^–/–^* cells (Fig. 4F, 4G). Similarly, the mRNA expression of *Lclat1* and *Lpiat* remained unchanged in deficient cells (Fig. 4H, 4I). These data indicate that ablation of *Abcb1* in macrophages alters PI lipid chain configuration independently of the synthesis of PI or remodeling of its acyl chain composition.

### Exogenous supplementation with LA alters PI acyl chain configuration

**Figure 5:**
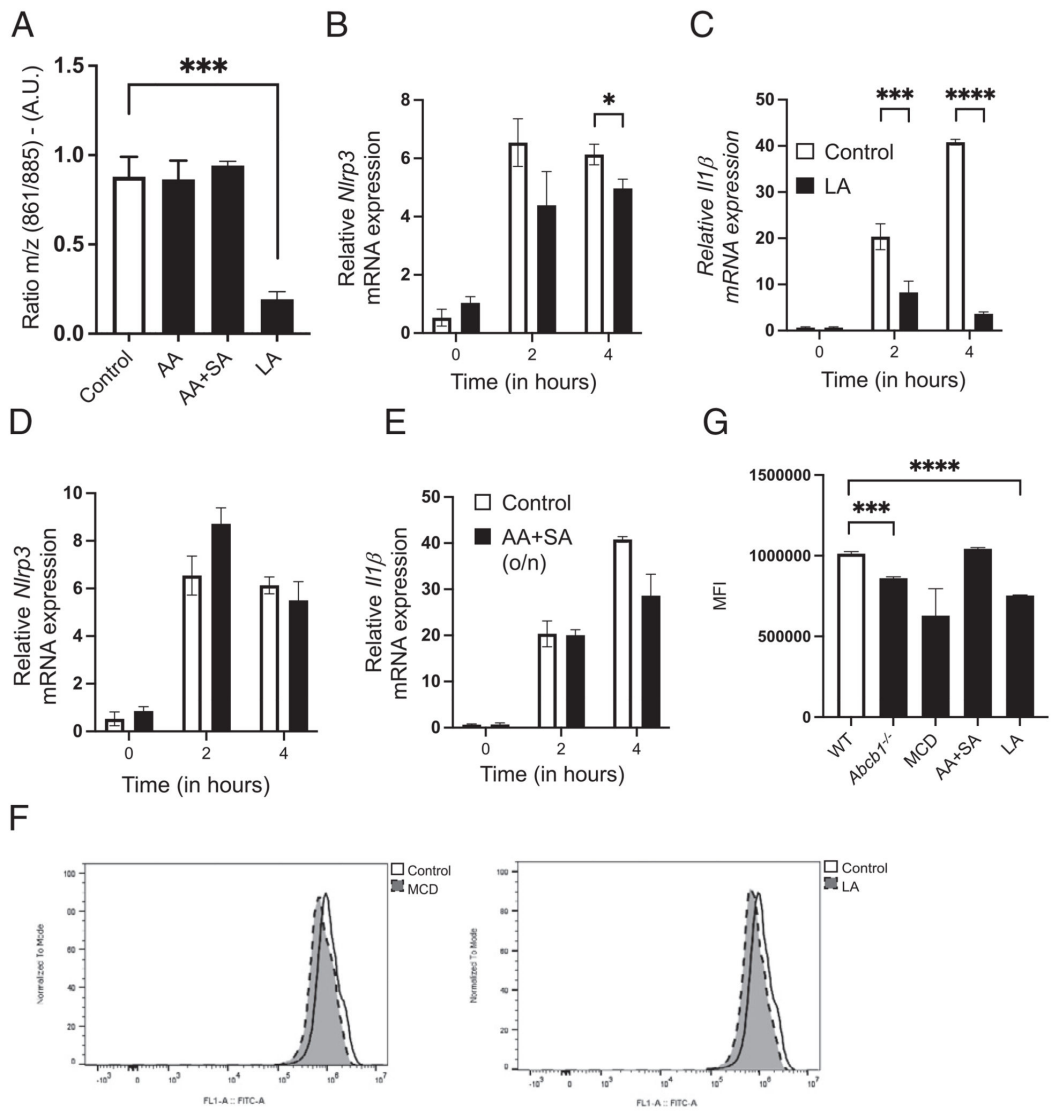
Exogenous supplementation with LA alters PI acyl chain configuration. WT cells were cultured in the presence of either AA (5 μM) or AA (5 μM) and SA (20 μM) or LA (20 μM) for at least 2 wk with the regular splitting of cell cultures every 2–3 d before subjecting the samples to whole-cell lipidomics. (**A**) Ratio *m/z* of peaks 861/885 in cells grown in the presence of indicated fatty acids. (**B–E**) WT cells cultured with either (B and C) LA or (D and E) AA and SA were treated with LPS (500 ng/ml) for the indicated time points, and the mRNA expressions of (B and D) Nlrp3 and (C and E) **Il1b** were analyzed. Gene expression is shown relative to Gapdh. (**F** and **G**) WT and *Abcb1* cells either untreated or treated with methyl-β-cyclodex-trin (10 μm, 30 min) or the indicated fatty acids were stained with CTB (1 μg/ml) for 10 min at 4°C followed by incubation with Alexa Fluor 488–conjugated anti-CTB Ab for 15 min at 4°C to reveal GM1 presence. (F) Fluorescence was analyzed by flow cytometry, and representative spectra are shown. (G) MFI (mean fluorescence intensity) quantification of cells treated as above. Data shown are mean ± SD and are representative of at least three independent experiments with three to five replicates each. *p ≤ 0.05, ***p < 0.001, ****p < 0.0001, by Student *t* test.

To further understand the mechanistic underpinnings of the shift in PI lipid chains in *Abcb1*-deficient cells, we next sought to identify the fatty acids, which may similarly alter PI acyl chains and subsequently modify inflammasome activity. In agreement with previous studies (27), we hypothesized that exogenous supplementation with distinct fatty acids will alter cellular PI acyl chain conformation. To achieve this, we grew cells for at least 2 wk in growth media supplemented with different fatty acids before validating the samples by MS (Fig. 5A). These assays revealed that prolonged supplementation with LA, but not AA, favored long-chain fatty acids (Fig. 5A, Supplemental Fig. 3). This was predominantly reflected in the increased concentration of unsaturated acyl chains at the *sn*-2 position. This demonstrates that cells supplemented with LA and those lacking ABCB1 both favor long-chain fatty acids, particularly the 885 species (Fig. 5A, Supplemental Fig. 3). Strikingly, supplementation with AA did not result in any noticeable difference compared with control cells (Supplemental Fig. 3C). Notably, we also observed a narrower range over which macrophages tolerated AA (data not shown). Accordingly, compared with control cells, WT cells grown in the presence of LA behaved similarly to cells lacking ABCB1 and demonstrated reduced *Nlrp3* and *Il1b* mRNA expression (Fig. 5B, 5C). Again, these results were specific for LA because supplementation with AA, alone or in combination with SA, neither altered the *m/z* 861/ 885 ratio nor the mRNA expression of *Nlrp3* and *Il1b* (Fig. 5A, 5D, 5E, Supplemental Fig. 3C). Furthermore, like *Abcb1^–/–^* cells, exogenous LA resulted in the disruption of GM1 presence in WT cells (Fig. 5F, 5G). These results therefore demonstrate that LA supplementation alters the PI acyl chain profile.

### PI acyl chain profile regulates TIRAP expression

TLRs are transmembrane receptors that can sense microbial products at distinct subcellular sites. Ligation of TLRs at both the plasma membrane and endosomes triggers a signal transduction pathway involving the adaptor protein MyD88, which is recruited to the conserved TIR domain present in the cytosolic tails of these receptors. Most TLRs recruit MyD88 by using the intermediate sorting adaptor TIRAP, which is bound to either phosphatidylinositol (4,5)-bisphosphate (PIP2) or phosphatidylinositol 4-phosphate for precise localization and is prepositioned on the membranes before TLR activation. The phosphorylation by Bruton’s tyrosine kinase activates TIRAP, but this is immediately followed by suppressor of cytokine signaling 1–mediated TIRAP polyubiquitination and degradation to avoid sustained signaling (40). The rapid turnover of TIRAP is similarly regulated by serine/threonine kinases and IRAKs IRAK1 and IRAK4, which directly phosphorylate TIRAP, triggering Lys48-linked ubiquitination and proteasomal degradation (41).

**Figure 6:**
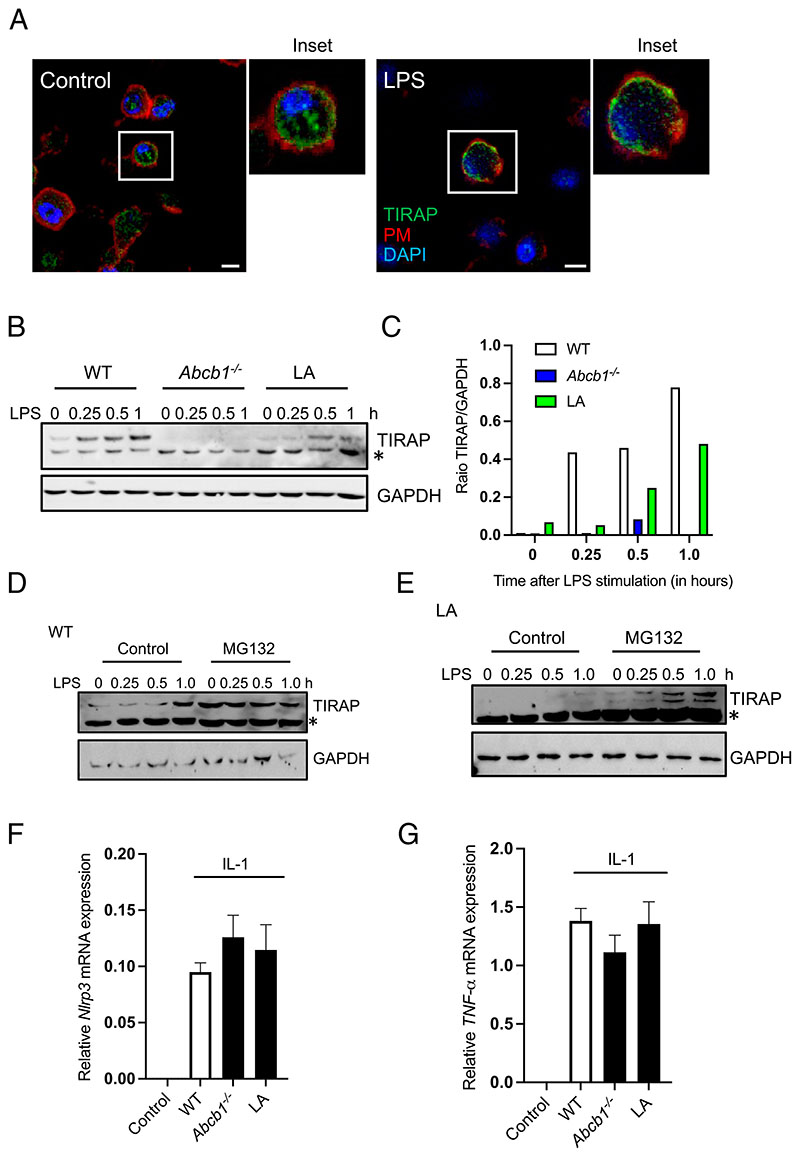
PI acyl chain profile regulates TIRAP expression. (**A**) Control and LPS-primed WT cells grown on coverslips were labeled with anti-TIRAP Ab, and nuclei were stained with DAPI. The plasma membrane was stained by adding Alexa Fluor 647–conjugated phalloidin for the last 15 min. (**B**) WT, *Abcb1^–/–^*, and LA-supplemented cells were stimulated with LPS for different times. Cell lysates were collected and immunoblotted for TIRAP and GAPDH. (**C**) Quantitation of the WB shown in (B) by ImageJ. (**D** and **E**) WT (D) and LA-grown (E) cells were pretreated or not with the proteasomal inhibitor MG132 (10 μM) for 30 min before stimulating the cells with LPS for different time points. Cell lysates were collected and immunoblotted for TIRAP and GAPDH. (**F** and **G**) WT, *Abcb1b^–/–^*, and LA-supplemented cells were exposed to IL-1 (2 ng/ml) overnight. RNA was extracted and converted into cDNA. Gene expression of *Nlrp3* and TNF-α was determined by real-time PCR. The data shown are representative of at least three independent experiments with three to five replicates each. Asterisk (*) on immunoblots denotes a nonspecific band. Scale bars, 5 μm.

A previous study demonstrated a critical role for PIPs in binding to TIRAP, and therefore maintaining its localization and stability (42). In the absence of PIP binding, TIRAP was found ubiquitinated followed by its degradation, thereby ablating the downstream TLR signaling. Therefore, we next evaluated whether the shift observed in PI-acyl chain composition affected TLR signaling by regulating TIRAP expression. As reported in previous studies, we mostly found TIRAP at the cell periphery with additional punctate staining in the cytoplasm (Fig. 6A). Stimulation of WT cells with LPS resulted in increased proximity of TIRAP to the plasma membrane (Fig. 6A). LPS stimulation resulted in a modest increase in TIRAP expression as observed by Western blot (WB) at 15 min followed by sustained TIRAP expression up to 60 min, the last time point that we tested (Fig. 6B, 6C). By contrast, cells lacking *Abcb1* or those exposed to LA exhibited significant depletion in TIRAP expression (Fig. 6B, 6C). After phosphorylation, TIRAP is degraded by the 26S proteasome. Accordingly, pretreatment of WT cells with the proteasomal inhibitor MG132 resulted in elevated TIRAP expression (Fig. 6D). In agreement, exposure of LA-supplemented cells to MG132 restored TIRAP expression (Fig. 6E). Having established the depletion of TIRAP in *Abcb1^–/–^* and LA-enriched cells, we next examined signaling from non-TLR stimuli, which function independently of TIRAP to activate the NF-κB pathway. Exposure to IL-1 overnight resulted in an increase in NLRP3 and TNF-α expression in WT cells (Fig. 6F, 6G). However, *Abcb1^–/–^* and LA-enriched cells showed comparable increase in expression on IL-1 stimulation (Fig. 6F, 6G). Notably, the increase in expression was several folds less compared with induction by LPS (data not shown). These studies therefore suggest that change in PI fatty acid configuration modifies TLR signaling by regulating the degradation of adaptor protein TIRAP.

### Altered PI acyl chain profile blunts inflammasome activity by increasing NLRP3 phosphorylation

**Figure 7:**
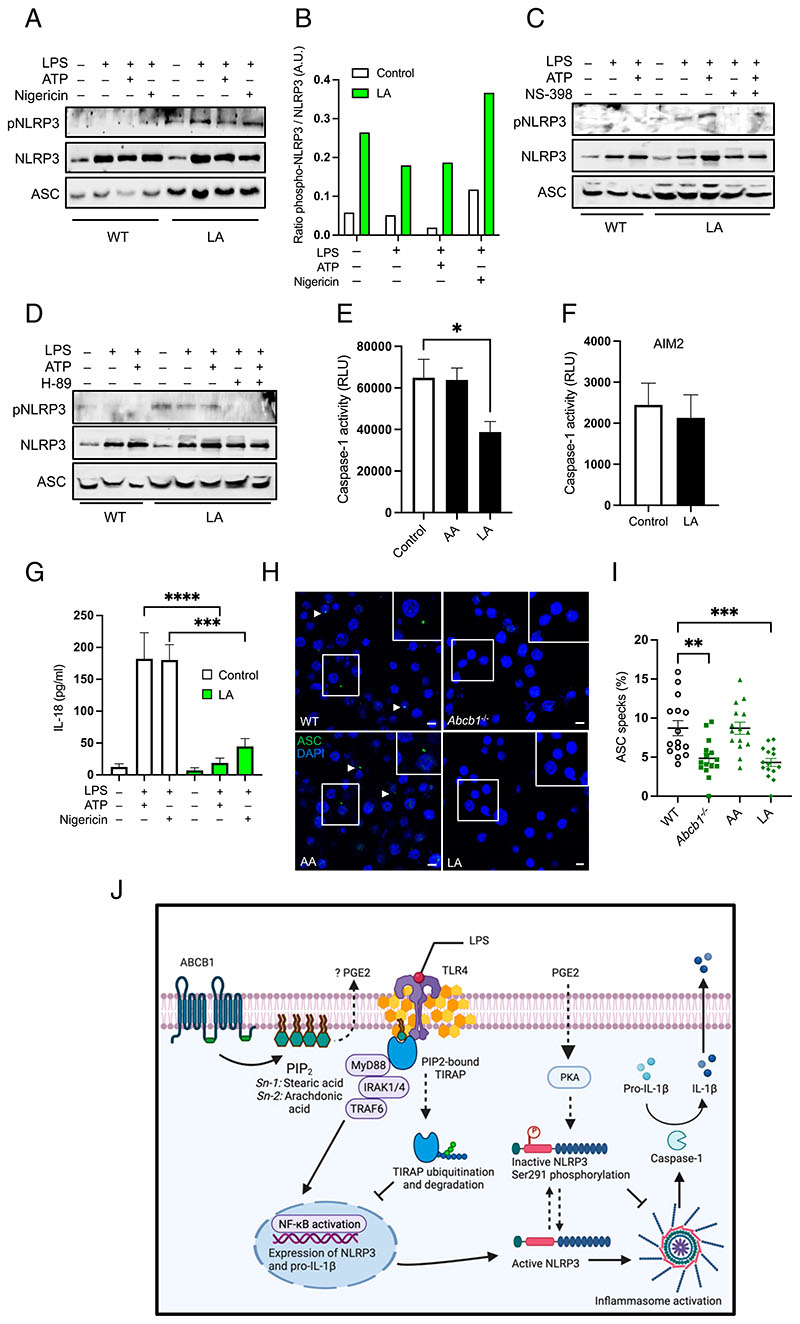
Altered PI acyl chain profile blunts inflammasome activity by increasing NLRP3 phosphorylation. (**A**) WT and LA-supplemented cells were either left untreated or primed with LPS (500 ng/ml) for 4 h followed by treatment with either ATP (5 mM) or nigericin (20 μM) for ~45 min. Cell lysates were collected, and protein was quantified. The amount of protein loaded for LA-supplemented cells was increased to normalize total NLRP3 levels. Cell lysates were immunoblotted for p-NLRP3, total NLRP3, and ASC. (**B**) Quantitative analysis of the WB shown in (A) by ImageJ. (**C** and **D**) Cells were treated with LPS and ATP and loaded as in (A). In addition, LA-supplemented cells were either exposed to COX2 inhibitor, NS-398 (10 μM), or PKA inhibitor, H-89 (10 μM), for overnight. Cell lysates were immunoblotted for p-NLRP3, total NLRP3, and ASC. (**E** and **F**) Control WT, AA-grown cells, and LA-grown cells were either primed with LPS (500 ng/ml) for 4 h followed by treatment with ATP (5 mM) for ~45 min or primed with LPS followed by transfection with AIM2 agonist, poly(dA:dT). Caspase-Glo 1 activity was measured in the culture supernatants. (**G**) Cell supernatants collected as in (A) were assayed for IL-18 secretion by ELISA. (*H*) WT, *Abcb1^–/–^,* AA-grown, or LA-grown cells cells were exposed to LPS + ATP followed by labeling with anti-ASC Ab and DAPI staining. Scale bars, 5 μm. (**I**) Quantitative analysis of the percentage of cells with ASC specks in samples treated as earlier. Each dot represents an individual field with at least *n* = 40 cells. Data shown are mean ± SEM, and the experiments shown are representative of at least three independent experiments with three replicates each. Arrowheads show ASC specks. *p < 0.05, **p ≤ 0.01, ***p < 0.001, ****p < 0.0001, by Student *t* test. (**J**) Schematic for ABCB1- and PI-mediated regulation of the NLRP3 inflammasome. ABCB1 is important in maintaining lipid metabolism. In the absence of ABCB1, the PI lipid chain configuration is altered, resulting in the reduced ratio of short-chain to long-chain fatty acids. The activation of the TLR4-dependent pathway relies on the adaptor protein TIRAP binding to PIP_2_ for precise positioning at the plasma membrane. However, alteration in PI-lipid profile results in at least two distinct outcomes, which affect both the priming and activation steps of the NLRP3 inflammasome. First, either due to inability to bind to PIP_2_ or reduced PIP_2_ abundance at the PM, TIRAP is ubiquitinated and degraded in the cytoplasm. Inflammasome assembly requires NLRP3, ASC, and pro-caspase-1 in a complex wherein caspase-1 activation leads to the maturation of pro-IL-1β into the activation form. Altered PI profile most likely increases PGE_2_ secretion because of increased PI incorporation of precursor AA. As a result, NLRP3 is phosphorylated at the Ser291 residue, which is mediated by PKA signaling leading to NLRP3 inactivation. Consequently, assembly of the inflammasome is abrogated resulting in blunted caspase-1 activation and IL-1β secretion.

The NLRP3 inflammasome is regulated both at the transcriptional and the posttranslational levels. In particular, NLRP3 phosphorylation at distinct sites regulates both the priming and activation steps. Notably, the LA derivative, PGE_2_, has been shown to facilitate NLRP3 phosphorylation in the NACHT domain at Ser291 position (Ser295 in humans) by inducing PKA and thus abolishing NLRP3 activation (43). Therefore, we next tested the status of NLRP3 Ser291 phosphorylation in control and LA-supplemented cells. We noted in preliminary experiments that cells exposed to LA exhibit, on average, 1.5 times less NLRP3 expression because of defective priming under these conditions (Supplemental Fig. 4G). To ensure that we examined phosphorylation on equal NLRP3 protein levels, we increased the amount of protein loaded onto gels in LA-supplemented cell lysates. Accordingly, increasing the protein concentration by the earlier fraction resulted in similar total NLRP3 expression in both control and LA-supplemented samples (Fig. 7A). By contrast, compared with control cells, LA-exposed cells demonstrated increased phosphorylation as examined using a p-NLRP3-specific Ab (Fig. 7A, 7B). We next sought to investigate how LA enrichment influenced NLRP3 phosphorylation. To address this, we examined the role of PGE_2_, an LA derivate, by impeding its production through inhibition of the upstream COX2, the rate-limiting enzyme in PGE_2_ biosynthesis. Exposure of LA-supplemented cells to the highly selective COX2 inhibitor, NS-398, ablated NLRP3 phosphorylation (Fig. 7C). Previous studies have shown that NLRP3 Ser291 phosphorylation is mediated by PGE2-induced PKA (43). In agreement, exposure of LA-supplemented cells to H-89, a potent PKA inhibitor, similarly ablated NLRP3 phosphorylation (Fig. 7D). These data demonstrate that altered PI acyl chain configuration results in elevated PGE_2_/PKA signaling, which phosphorylates NLRP3 and blunts inflammasome activation.

We next assessed caspase-1 activation in LA-supplemented cells and found that they phenotypically mimic *Abcb1^–/–^* cells. Cells supplemented with LA and exposed to NLRP3 stimuli demonstrated reduced caspase-1 activity, which remained similar in cells exposed to AA (Fig. 7E). However, in agreement with data in *Abcb1^–/–^* cells (Fig. 3), LA supplementation did not alter caspase-1 activity on AIM2 inflammasome activation (Fig. 7F). Moreover, LA supplementation diminished the secretion of IL-18 from LPS-primed cells in response to both ATP and nigericin (Fig. 7G). To further validate our results, we next examined inflammasome assembly by examining ASC speck formation. NLRP3 inflammasome activation by LPS + ATP resulted in a comparable percentage of ASC specks in both control WT and cells exposed to AA (Fig. 7H, 7I). By comparison, cells lacking *Abcb1* exhibited significantly reduced ASC specks. Similarly, cells grown in LA-rich media displayed reduced ASC speck formation (Fig. 7H, 7I). Because LA supplementation also affected NLRP3 expression, the contribution of priming to caspase-1 activation and ASC speck formation cannot be excluded in these experiments. Nevertheless, together with the elevated NLRP3 phosphorylation observed on LA sup-plementation, these data demonstrate that altered PI acyl chain configuration affects both the priming and activation steps of the NLRP3 inflammasome.

## Discussion

Our studies described in this article demonstrate that lipid metabolism is intricately linked to immune signaling and inflammasome activation. Genetic or pharmacological depletion of ABCB1 abolished caspase-1 cleavage and IL-1β secretion. Remarkably, this was restricted to the NLRP3 inflammasome be-cause *Abcb1^–/–^* cells displayed comparable activation of the NLRC4 and AIM2 inflammasomes. Further mechanistic studies based on whole-cell lipidomics revealed an altered PI lipid chain profile in deficient cells. Remarkably, modified PI lipid chain configuration accompanied reduced TIRAP expression and altered NLRP3 phosphorylation; together they contributed both to NLRP3 inflammasome priming and activation steps (Fig. 7J). Notably, prolonged growth in media supplemented with LA reconfigured the PI acyl chain profile in WT cells, thereby mimicking the phenotypic features observed in deficient cells, including aberrant inflammasome activation. Our results therefore demonstrate the metabolic regulation of inflammasome activation by PI acyl chains.

PIPs, which take key roles in membrane trafficking and signal transduction, are defined by the phosphorylation of their inositol head group (44, 45). By contrast, the significance of the attached fatty acids has remained unappreciated (39, 46). In mammalian cells, ~40-80% of the PIP molecules contain stearoyl/arachidonyl in the *sn*-1 and *sn*-2 positions, respectively, designated as C18:0/C20:4 or 38:4 for the complete molecule (45). A previous study revealed that mutations in the *p53* gene expand PIs containing short-length acyl chains, corresponding to 36-carbon PIs (47). Remarkably, the shift in PI-lipids was not reflected in the lipid spectra of phosphatidylcholine (PC), asserting that the modification is not distinctive of all phospholipids (47). Notably, both PC and PI share a common precursor, PA. However, the latter steps in the pathway involve distinct enzymes to generate either PI or PC. Our studies revealed that *Abcb1*-deficient cells expressed comparable levels of PI synthase, as well as the enzymes involved in PI acyl chain remodeling, thus excluding these as a probable reason. As a result, it remains unclear as to how *Abcb1*-deficient cells acquire a distinct composition of PI-lipid chains. It is tempting to speculate that the precursor PA possessing a different lipid content gets accumulated in distinct membranes, which is spatially only accessible to PI enzymes in deficient cells. Alternatively, it is also possible that the mature PIs are able to remodel their lipid chains once they have formed. If true, it will be interesting to decipher the mechanisms that prompt this variation.

Our study suggests that ABCB1 is critical in retaining immune equilibrium, and the loss of ABCB1 dampens inflammasome activation. Keeping in view that aberrant PI signaling (including by the PI3K/AKT pathway) is associated with malignancies, it is plausible that ABCB1 does not exclusively regulate the PI-lipid profile, and there exists redundancy in this pathway acting as a fail-safe mechanism. Accordingly, supplementation with LA similarly altered the PI profile incorporating elevated AA levels. Notably, this phenomenon did not occur in cells directly exposed to AA. AA has been shown to exhibit potent proapoptotic effects on macrophages by causing cell-cycle arrest (48). In agreement, we observed a limited range in which macrophages tolerated AA (data not shown). LA is the most highly consumed polyunsaturated fatty acid found in the human diet. It is also the parent compound for the family of v6 polyunsaturated fatty acids, including AA, which is further converted to a range of bioactive compounds such as leukotrienes, PGs, and eicosanoids. Together, this suggests that LA is the preferred pathway for AA expansion at the PI *sn*-2 position.

The activation of effector responses on TLR ligation relies on signaling cascades involving TIRAP/MyD88 or TRAM/TRIF adaptor molecules, which activate NF-κB or IFN-dependent signaling. Our data revealed that modified PI-lipid chains elicit rapid degradation of the adaptor protein TIRAP. Bound to phosphatidylinositol 4,5-bisphosphate–enriched plasma membrane regions or phosphatidylinositol 3-phosphate at the endosomes, TIRAP surveys these compartments for activated TLRs. Notably, interaction with PIPs at its N-terminal PIP-binding domain is critical for TIRAP membrane recruitment and retention. A recent study demonstrated the participation of basic and nonpolar residues in the TIRAP PIP-binding domain. Significantly, the authors found both the inositol head group and acyl chains as critical in binding to TIRAP (42). Under conditions of reduced PIP binding, IRAK1/4 phosphorylated Thr28 residue within the PIP-binding motif leading to TIRAP ubiquitination and degradation (42). Independently, another study demonstrated that the ratio of PIP_2_ to PIP_3_ at the plasma membrane influenced TLR signaling (49). A decrease in PIP_2_ abundance at the plasma membrane concurrently resulted in TLR4 internalization and TIRAP redistribution to cytoplasmic compartments, where it was degraded by the proteasome and calpain. Altogether, it is tempting to speculate that the modified PI is either incompetent in TIRAP binding and/or fails to localize to the plasma membrane, resulting in TIRAP ubiquitination and degradation.

NLRP3 inflammasome is regulated both at the transcriptional and the posttranslational levels. In particular, NLRP3 phosphorylation at distinct sites may either activate or inhibit the inflammasome. We observed that the increased incorporation of long-chain AA by PI in WT cells through LA supplementation resulted in Ser291 phosphorylation in the NACHT domain. Previous studies have shown increased phosphorylation at this site (Ser295) in THP-1 cells as a result of PKA activation by PGE_2_, resulting in dampened inflammasome activation (43). Considering that linoleic and AA are both precursors to PGs, it may be interesting to test the overall basal levels of PGs in these cells. Nevertheless, our studies reveal the regulation of NLRP3 inflammasome by PI synthesis and metabolism.

Immune signaling triggers an adjusted inflammatory response, and any overt activation of these pathways may result in collateral damage. Therefore, it needs to be calibrated by mechanisms that offer a variable degree of responses and are infallible. Although the phosphorylation of the inositol head group may at most result in seven distinct PIP species, the possibility to add an array of fatty acids, with different carbon lengths and saturation statuses, at the two positions on the PI glycerol linker offers far greater flexibility in terms of functions the cellular PIs can serve. In conclusion, our study provides insights as to how changes in PI lipid profile modify inflamma-some activity and advance our understanding of the cross-talk between lipid metabolism and immune signaling.

## Supplementary Material

Supplementary Figures

## Abbreviations used in this article

AA: arachidonic acid
ABCB1: ATP Binding Cassette Subfamily B Member 1
ASC: apoptosis-associated speck-like protein containing a CARD
BMDM: bone marrow–derived macrophage
CDP-DAG: cytidine diphosphate-diacylglycerol
COX2: cyclooxygenase 2
CTB: cholera toxin subunit B
gRNA: guide RNA
GSDMD: gasdermin-D
iBMDM: immortalized bone marrow-derived macrophage
IRAK: IL-1R–associated kinase
LA: linoleic acid
MOI: multiplicity of infection
MS: mass spectrometry
m/z: mass to charge ratio
NACHT: NAIP, CIITA, HET-E, and TP-1
NLRP3: NOD-like receptor family pyrin domain–containing 3
PA: phosphatidic acid
PC: phosphatidylcholine
PI: phosphatidylinositol
PIP: phosphoinositide
PIP2: phosphatidylinositol (4,5)-bisphosphate
PKA: protein kinase A
poly(dA:dT): poly(deoxyadenylic-deoxythymidylic) acid sodium salt
Rho123: rhodamine-123
SA: stearic acid
TIRAP: Toll/IL-1R domain–containing adaptor protein
WB: Western blot
WT: wild-type
