# Supplementary material for: NLRP3 Inflammasome Priming and Activation Are Regulated by a Phosphatidylinositol-Dependent Mechanism
Source: Immunohorizons. Author manuscript; Available in PMC 2022 Sep 7. (PMC7613559; doi:10.4049/immunohorizons.2200058)
Supplement: Supplementary Figures [file EMS153594-supplement-Supplementary_Figures.pdf]

A

Gene	Forward Primer	Reverse Primer
Gapdh	CGTCCCGTAGACAAAATGGT	TTGATGGCAACAATCTCCAC
Nlrp3	TCACAACTCGCCCAAGGAGGAA	AAGAGACCACGGCAGAAGCTAG
Il1β	GATCCACACTCTCCAGCTGCA	CAACCAACAAGTGATATTCTCCATG
Tnfα	CATCTTCTCAAAATTCGAGTGACAA	TGGGAGTAGACAAGGTACAACCC
KC	CAATGAGCTGCGCTGTCAGTG	CTTGGGGACACCTTTTAGCATC
Ifnβ	GCCTTTGCCATCCAAGAGATGC	ACACTGTCTGCTGGTGGAGTTC
Abcb1a	AACTCAGAGCCGCTTCTTCC	CTGTCCAGCCAACCTGCATA
Abcb1b	GGTGGTGTCATTGTGGAGCAAG	GCATCAGTGTCACTCTGGGATC
Abcg2	CAGTTCTCAGCAGCTCTTCGAC	TCCTCCAGAGATGCCACGGATA
Lpiat	TGCTATGGCACAGACTTCTGCG	ACTCAACAGCATGGTCCAGGCA
Lclat1	CCAGAAGGAACTGACCTCACAG	TCTCTTAGGCGGTCCACCACAA
Cdipt	GGACTCCTAGACGCTTTCGATG	CAGGTTGACCAAGAGACACATGG
Cds2	GGCTTCTTTGCCACTGTGGTGT	CCACAGTGAAGCTGTTGGTGTC

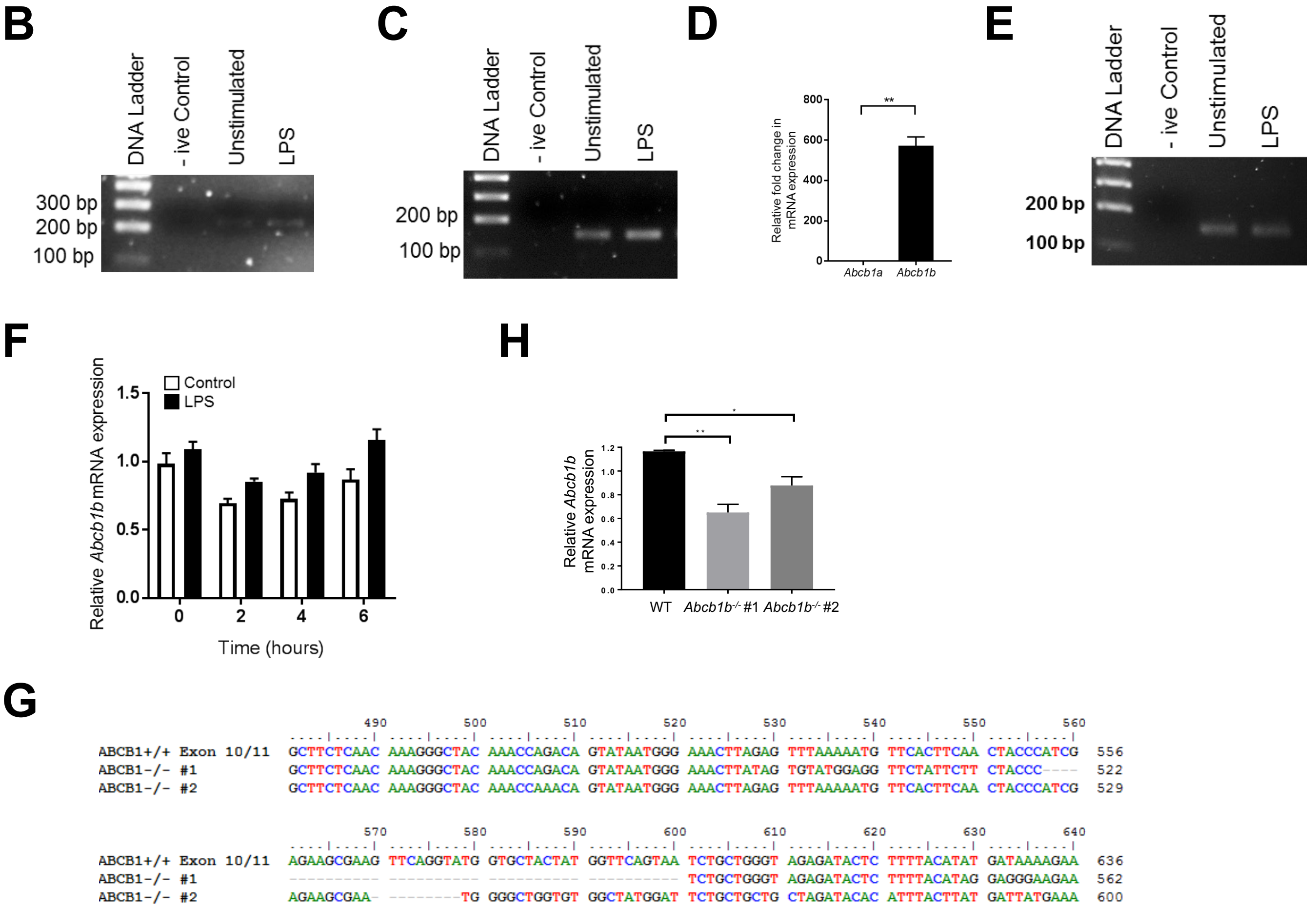

Supplementary Figure 1. (A) Sequence of PCR primers used in the study. **(B)** ABCB1 gene expression in mouse macrophages. cDNA from control and LPS-primed (4 hours) immortalised BMDMs was amplified using primers specific to **(B)** *Abcb1a* or **(C)** *Abcb1b*. PCR products were separated by gel electrophoresis. **(D)** Relative fold change in the mRNA expression of *Abcb1a* and *Abcb1b* mRNA expression in iBMDMs by real-time PCR. mRNA expression is shown relative to *Gapdh*. **(E)** Primary mouse BMDMs were analysed for *Abcb1b* gene expression by PCR and separated by gel electrophoresis. **(F)** WT iBMDMs were stimulated with or without LPS (500 ng/ml) for the indicated time points and analysed by real-time PCR for *Abcb1b* mRNA expression. **(G)** Lentiviral particles containing sgRNA against *Abcb1b* exons 10 and 11 were generated and transduced into iBMDMs. Single-cell colonies were then generated, and clonal cell lines were assessed for deletions in exon 10 and 11 of the *Abcb1b* gene by Sanger sequencing. Two clonal lines with mutations were identified (*Abcb1b*$^{-/-}$ #1 and #2). **(H)** *Abcb1b* mRNA expression from CRISPR/Cas9 *Abcb1b*$^{-/-}$ macrophages generated in B. Data shown are mean ± SD and is representative of at least three independent experiments. *, p=<0.05, ** p = <0.01, by Student's t-test.

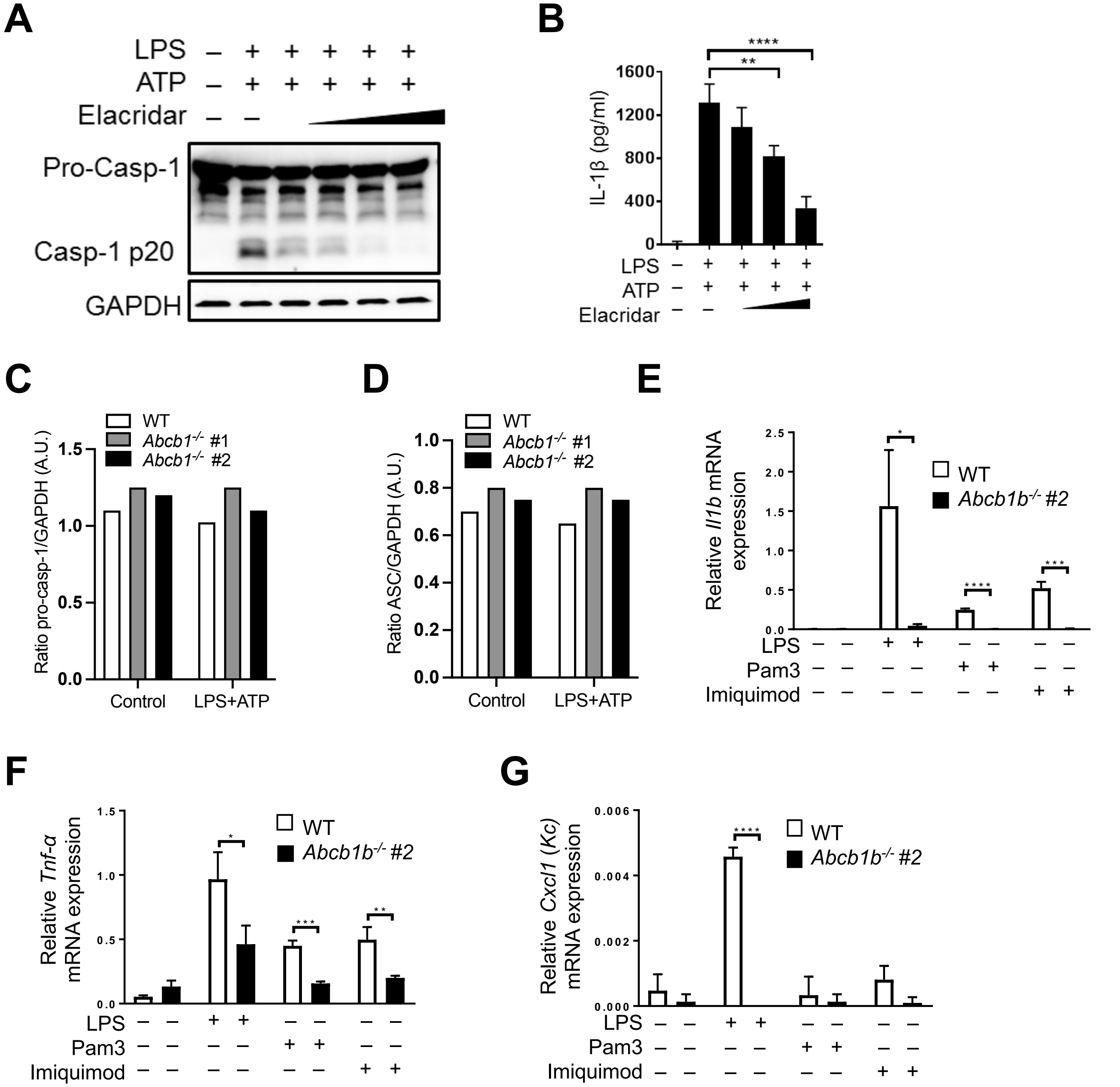

Supplementary Figure 2. ABCB1 regulates NLRP3 inflammasome. (A) WT BMDMs were treated with increasing amounts of elacridar overnight (1, 2, 5, and 10 µM), followed by LPS priming (500 ng/ml) for 4 hours and ATP (5 mM) for approximately 45 minutes. Cell lysates were immunoblotted for caspase-1 and GAPDH. **(B)** Supernatants from cells treated as in A were analysed for IL-1β by ELISA. **(C,D)** Densitometry analysis of pro-caspase-1 and ASC from blots shown in Figs. 1F and 2A in WT, *Abcb1b*$^{-/-}$ #1, and *Abcb1b*$^{-/-}$ #2 cells. WT and *Abcb1b*$^{-/-}$ #2 cells were stimulated with either LPS (500 ng/ml), Pam3 (1 µg/ml) or imiquimod (1 µg/ml) for 4 hours and analysed for mRNA expression of **(E)** *Il1b,* **(F)** *Tnfα,* and **(G)** *Cxcl1 (KC).* mRNA expression shown is relative to *Gapdh*. Data shown are mean ± SD, and experiments shown are representative of 3 independent experiments. n.s, not significant, *, p = <0.05; **, p = <0.01; ***, p = <0.001; ****, p = <0.0001, by Student's t test.

A

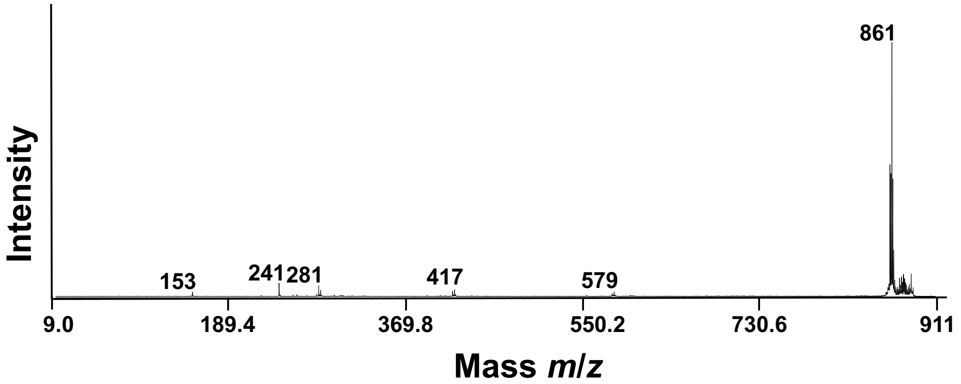

B

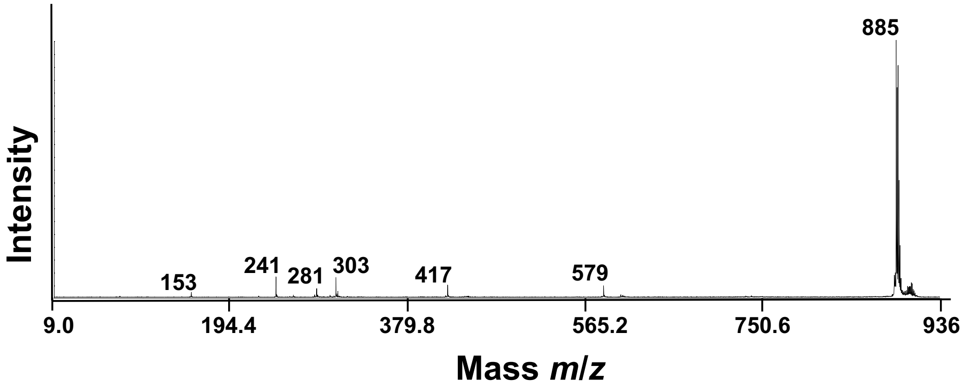

C

Control	Mass (*m/z*)		Dominant molecular species	Mean total iBMDM PI (%)
	821	33:1		5.57
	835	34:1	16:0/18:1	7.96
	861	36:2	18:1/18:1	39.75
	885	38:4	18:0/20:4	45.53
**Arachdonic acid**				
	835	34:1	16:0/18:1	6.72
	861	36:2	18:1/18:1	38.29
	885	38:4	18:0/20:4	45.01
**Linoleic acid**				
	835	34:1	16:0/18:1	3.26
	861	36:2	18:1/18:1	12.99
	885	38:4	18:0/20:4	69.01

Supplementary Figure 3. MS/MS mass spectra of peaks at *m/z* 861 (A) and *m/z* 885 (B). The fragment at *m/z* 241 corresponds to the polar head group fragment assigned to as inositol phosphate minus water. The fragment at *m/z* 153 corresponds to glycerophosphate minus water. The fragment at *m/z* 281 represents the fatty acid anions of oleic acid. In spectra A, the fragment ion at *m/z* 579 indicate the neutral loss of 282 corresponding to oleic acid and the fragments ions at *m/z* 417 show an additional neutral loss of 162 corresponding to an inositol unit minus water from the lyso-PI fragment ion at *m/z* 579. In spectrum B, the fragment ion at *m/z* 579 indicates the neutral loss of 306 corresponding to an eicosadienoic acid and the fragment ion at *m/z* 417 shows an additional neutral loss of 162 corresponding to an inositol unit minus water from the lyso-PI fragment ion at *m/z* 579. The fragment at *m/z* 303 represents the fatty acid anion of eicosatetraenoic acid. **(C)** The relative levels of total iBMDM percent PI content in cells either untreated or exposed to indicated fatty acids. Cells were either left untreated or exposed to arachidonic acid or linoleic acid for at least two weeks before whole-cell lipidomics was carried out. The ratio of a particular PI species to the whole-cell representation of all observed PI species was calculated and shown here in percentage.

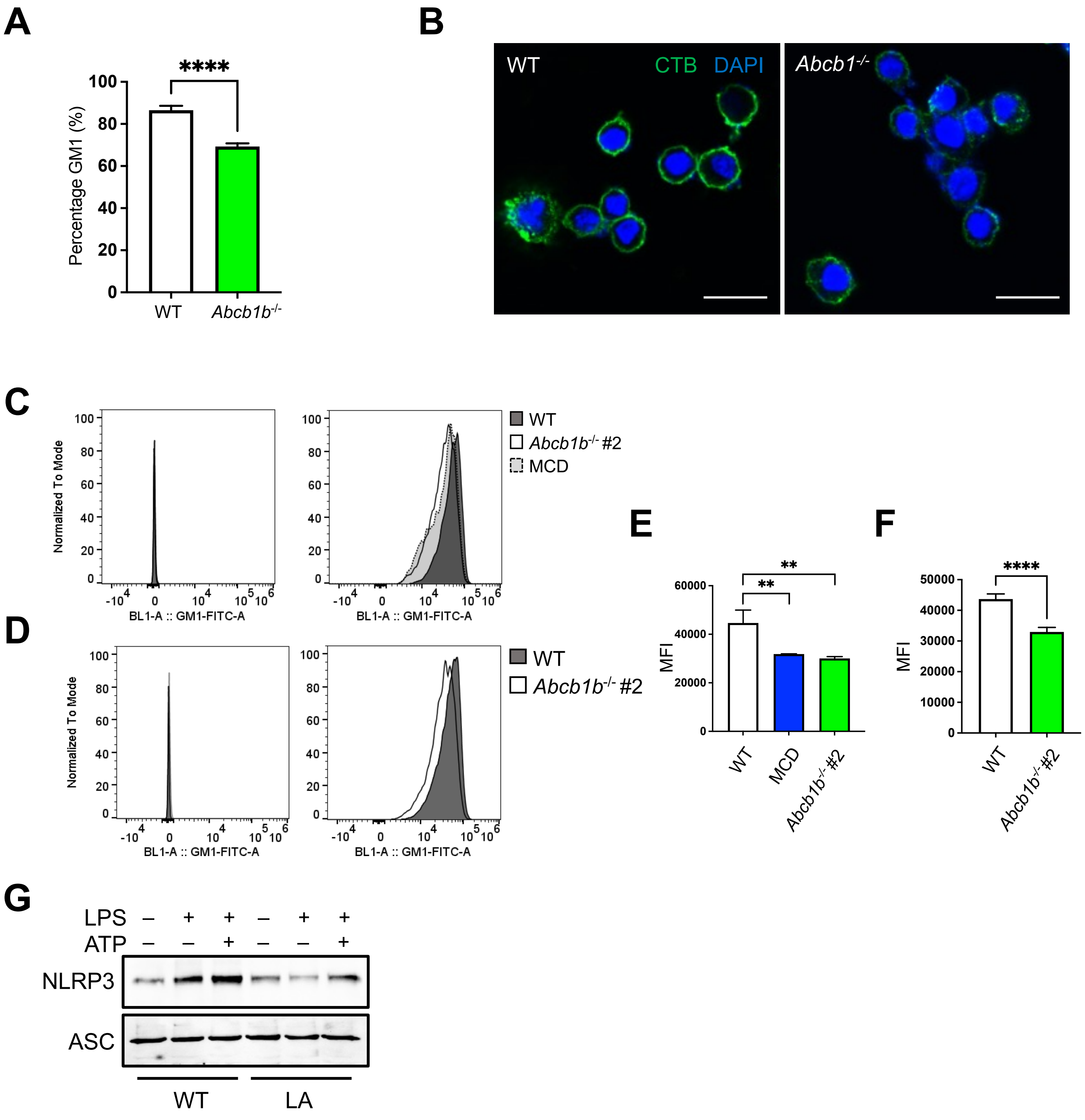

Supplementary Figure 4. ABCB1 deficiency disrupts GM1-positive membrane microdomains. (A) Percentage GM1 as a total of GM1 and GM2 peaks identified in Fig 4A. **(B)** WT and *Abcb1b*$^{-/-}$ cells grown on coverslips were stained with cholera toxin B (CTB) (1 µg/mL) for 10 minutes at 4 °C followed by incubation with Alexa Fluor 488-conjugated anti-CTB antibody for 15 minutes at 4 °C to reveal GM1 presence by confocal microscope. **(C)** WT cells either untreated or treated with MβCD (10 µm, 30 min) and *Abcb1b*$^{-/-}$ cells were stained with Cholera Toxin subunit B (CTB) as above. Fluorescence was analysed by flow cytometry and representative spectra are shown. **(D)** WT cells and *Abcb1b*$^{-/-}$ cells were stimulated with LPS (500 ng/ml) and stained with CTB as above. **(E)** MFI (Mean fluorescence intensity) quantification of cells treated as in C. **(F)** MFI (Mean fluorescence intensity) quantification of cells treated as in D. **(G)** WT and linoleic acid-supplemented cells (LA) were either left untreated or primed with LPS (500 ng/ml) for 4h or with LPS for 4h and followed by ATP (5 mM) for approximately 45 minutes. Cell lysates were collected, and protein quantified. Cell lysates were immunoblotted for total NLRP3 and ASC. Data shown are mean ± SD and is representative of at least three independent experiments. **, p=<0.01, **** p = <0.0001, by Student's t-test.
